# Estimating firm digitalization: A method for disaggregating sector-level digital intensity to firm-level

**DOI:** 10.1016/j.mex.2021.101233

**Published:** 2021-01-18

**Authors:** Tomasz Mucha, Timo Seppälä

**Affiliations:** a Department of Industrial Engineering and Management, Aalto University, Espoo, Finland; b Research Institute of the Finnish Economy, ETLA, Helsinki, Finland

**Keywords:** Digital transformation, Digital taxonomy, IT intensity, Data disaggregation

## Abstract

The digital transformation of firms plays an increasingly important role in the economy and society. However, limited access to data on firm-level digital intensity is an impediment to advancement of multiple research projects concerned with firm digitalization. To alleviate this challenge, this paper proposes a method for estimating firm-level digital intensity based on other more readily available firm-level data and reference data on digitalization, which is available on sector-level. The proposed method utilizes firm-level revenue breakdown by sector to estimate sector revenue-weighted digital intensity scores, which lead to classification of firms into low, medium and high digital intensity groups. The output from the proposed method can be directly used in research concerned with firm digitalization and investigating this multifaceted phenomenon. Results from the application of the proposed method to an illustrative sample of large US and non-US firms (2000 observations in total) indicate that firm-level digital intensity can be efficiently estimated for large samples using data commonly available to researchers.

The key differences between the proposed method and alternative methods are:

• Recognition of the fact that firms might participate in more than one sector or industry, which partially explains within-sector heterogeneity in firm-level digital intensity. We found that 67.8% of large US firms and 78.6% of large non-US firms were engaged in more than one industry.
• Use of reference sector-level digital intensity scores, which allows for rapid update, application across geographies and time, as well as parallel calculation of multiple digital intensity scores for each reference data. Furthermore, use of reference data enables supplementation of firm-level data on digitalization.
• Replicability of the method and reproducibility of the results through inclusion of the source code and availability of data through research and commercial databases.

## Graphical abstract

**Figure:**
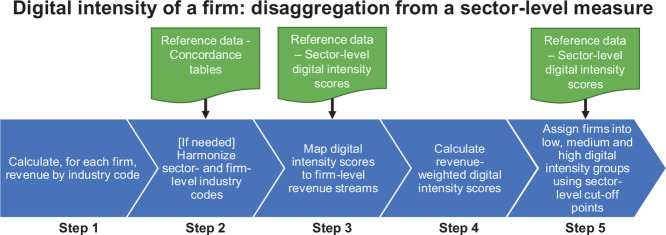

Specifications Table

**Table:** 

Subject Area:	Economics and Finance
More specific subject area:	Information Economics
Method name:	*Digital intensity of a firm: disaggregation from a sector-level measure*
Name and reference of original method:	Calvino, F., et al. (2018), "A taxonomy of digital intensive sectors", *OECD Science, Technology and Industry Working Papers*, No. 2018/14, OECD Publishing, Paris, https://doi.org/10.1787/f404736a-en
Resource availability:	Source code in R is available in the supplementary material for this article. Firm level-data available from annual reports of publicly listed companies or financial data bases. Sector-level digital intensity scores available, for example, from dx.doi.org/10.1787/888933617434. Industry classification concordance tables available, for example, from https://www.census.gov/eos/www/naics/concordances/concordances.html.

## Method details

The digital transformation of firms plays an increasingly important role in the economy and society. Digitalization affects organizations from a variety of angles and levels [1]. Furthermore, this phenomenon impacts organizations across the full range of industries and sectors [2]. Hence, research on digitalization of firms and other phenomena related to it is of significant importance to the society. This observation is supported by increasing research interest in these topics across various disciplines [3]. Such research is enabled, but also potentially limited, by the extent of available methodological toolbox. Methods used in research on digitalization span a wide range, including both quantitative and qualitative methods [1]. These methods take a variety of data as inputs, such as case studies [1], aggregate measures of investment in information and communication technologies (ICT) stock [4,5], purchases of intermediate ICT goods and services [6], robot use [7,8], online sales [6], and occupational classification and task-based index of digital intensity [9]. However, due to the fact that “inherent difficulties exist in measuring the scope and pace of such a multifaceted phenomenon” [6, p. 5] as digitalization, access to suitable data might be an impediment to advancement of our understanding.

The present paper proposes a method, which alleviates the challenge of insufficient firm-level data by leveraging suitable results from past research on sector-level digitalization. The proposed method utilizes firm-level revenue breakdown by sector to estimate sector revenue-weighted digital intensity scores. These scores are derived from existing results of research on sector-level digitalization. The method output is a classification of firms into low, medium and high digital intensity groups.

The reminder of this paper is divided into three sections. We first discuss input data. After that we describe steps in the method and conclude with method validation. The paper is accompanied with supplementary material, which includes R code for implementation and validation of the method, as well as sample data used in the validation section.

### Input data

The implementation of the proposed method relies on three categories of input data. First two are necessary, while the third one is used in special cases only. These categories are:

1. Firm-level data on revenue per sector or industry.
2. Reference sector-level digital intensity scores.
3. Additionally, in case these two categories of data listed above rely on different industry classification systems, there is a need for a concordance table, which maps industry classification codes on a firm-level to those on a sector-level.

#### Firm-level data

Firm-level data is the data describing companies of interest. At a minimum, firm-level data must include firm-specific identifier, industry or sector code (thereafter, referred to as industry code, for brevity) and corresponding revenue or share of annual revenue. A single company might be active in either one or many industries. Additional information, such as firm name and industry name is useful to include to facilitate manual inspection of data processing steps, when in the development phase. Once the proposed method produces its outputs, these intermediary results will likely need to be combined with other data and subjected to analysis to address specific research questions.

It is important to recognize that the proposed method uses, for each company, revenue figures allocated to relevant industries as basis for calculating weights, which in turn are utilized to calculate revenue-weighted digital intensity score of each company. We motivate the use of revenue as the key determinant of industry participation with the following logic. Companies generating revenue from a given industry are likely to have characteristics similar to those of other companies in that industry. This is driven by similarity of the environmental conditions in which they operate, such as customer base, regulation, competition, technology context, etc. In summary, our argument for the use of revenue split by industry as a proxy for digital intensity score weights is based on the institutional isomorphism logic [10]. Thus, digital intensity of a company should, approximately, be the digital intensity of each industry where that company is active and proportional to the level of activity in these industries.

**Fig. 1:**
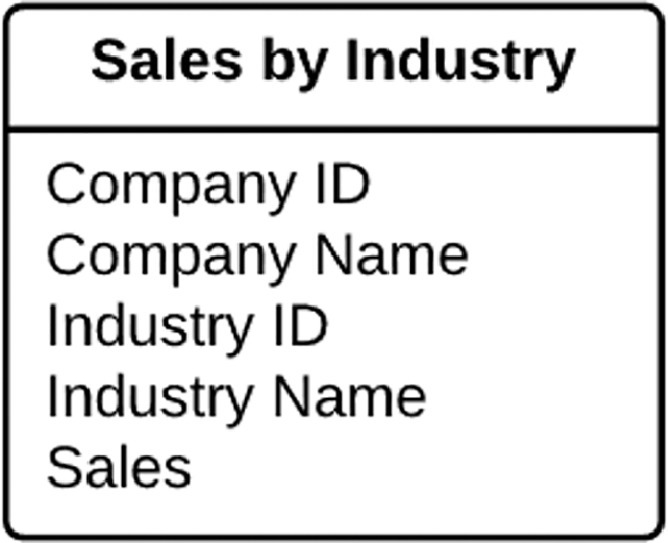
An example of a simple data structure for firm-level data.

While the firm-level data can take a simple format, as presented in the Fig. 1, it is common to encounter more complex input data and data issues. For example, there might be multiple industry codes grouped together and representing a single business segment of a company, which is accompanied by a single revenue figure. Another difficulty might be negative figures reported as eliminations resulting from inter-segment sales. Finally, industry classification systems have been periodically revised, thus it is possible to encounter industry codes from different revisions of an industry classification system listed in the same data set. We propose several sub-procedures for dealing with such data issues in the latter section of this paper. If other types of complexities are encounters, researchers must use common sense to process or convert the data to comply with the requirements of the latter steps in the procedure. Furthermore, any such judgement calls and additions to the procedure should be documented and reported.

#### Sector-level data

Sector-level data is the source data for digital intensity scores. Our method leverages previous research on the digital and IT intensity of industries, for example [6,11]. Published results for digital intensity of industries serves as a reference data for the proposed method. This approach presents some limitations, which need to be recognized before application of the method. Scholars applying the proposed method in own research ought to assess the suitability of the sector-level reference data for the estimation of digital intensity on a firm-level for the specific sample of companies under investigation. Researchers need to evaluate the alignment between the two data sets considering multiple factors. First, the alignment in time frame needs to be assessed. Since digital intensity of sectors might be changing over time [6], it is important to evaluate whether the reference data is representative of the sample, given potential temporal changes in digital intensity. Next, there are differences in the level of sectoral digital intensity in different countries [6], thus overlap in geographic coverage needs to be considered. Firm size is another important aspect, as size is positively correlated with variables associated with digital technology adoption [4]. These variables include, but are not limited to, slack resources, access to finance, wealth, scale, and specialization [12,13]. Another set of factors relate to market concentration and competitiveness, which can be assessed, for example, using Herfindahl-Hirschman index [14]. Market concentration and competitiveness are associated with adoption rates for high technology [12,15], thus alignment between the reference data and the sample data needs to be assessed with this respect as well. Finally, the methodology used in the sector level analysis leading to the reference data should be evaluated for suitability with the research question at hand. Other factors potentially undermining the suitability of the reference data for use with the specific sample under investigation might need to be considered as well. Yet, given limited availability and difficulty with access to information needed for calculating digital intensity directly on a firm-level, use of a reference data on a sector-level presents a viable alternative. Furthermore, this approach enables researchers to estimate on a per-firm basis multiple digital intensity scores based on alternative reference data sources, as well as efficiently revise existing digital intensity scores when new reference data becomes available.

**Fig. 2:**
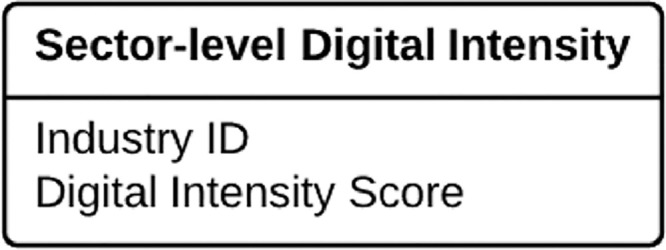
The simplest possible format for a sector-level digital intensity score.

Sector-level digital intensity data takes the form of a simple look-up table with industry codes and their respective digital intensity scores, as presented in Fig. 2. It is useful to retain industry names in the data to facilitate debugging of the procedure, while in the development phase. Potential complexities relate to the aggregation of multiple industries into ranges of industry codes. This might also be associated with some papers using industry codes on different levels in the taxonomy of an industry classification system. While simple aggregation of industries based on industry taxonomies are straightforward to handle, researchers developing sector-level digital intensity scores might also make discretionary decisions regarding aggregation into higher-level industries or sectors. In such cases it is important to evaluate and, potentially, disentangle earlier modifications to the industry classification taxonomy. Again, transparency and common sense need to be applied and choices documented.

#### Concordance table

**Fig. 3:**
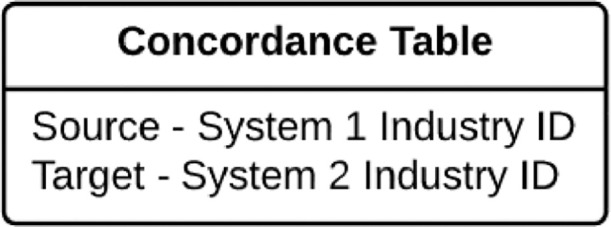
Data structure in a concordance table.

According to U.S. Census Bureau, concordance tables “provide detailed descriptions of the direct relationships between classification systems” [16]. These tables map industry codes from one industry classification system to another, as well as map industry codes within the same classification system for different revisions of that system. The data structure for concordance tables is presented in Fig. 3.

In cases where the firm-level data or both firm- and sector-level data include industry codes from different industry classification systems or different revisions of the same system the use of concordance tables will be required in the application of the proposed method. Concordance tables are provided by national or international census or statistical offices and, therefore, tend to be a reliable, replicable and easily available. However, potential data issues might relate to translation of older industry classification systems into more recent ones. For example, U.S. Census Bureau does not provide direct concordance table between NAICS (North American Industry Classification System) 1997 to NAICS 2017. In the next section of this paper we discuss two approaches for dealing with such data issue.

### Steps in the method for disaggregating sector-level digital intensity scores to firm-level

In this section, we first outline the steps involved in the implementation of the proposed method. Thereafter, we discuss each step and provide a commentary on how to deal with potential data issues.

The key steps in the implementation of the method are:

1. For each company retrieve data with or calculate revenue figure for each industry code.
2. In case firm-level and sector-level data uses different industry classification systems or different revisions of the same classification system, use concordance table(s) to convert firm-level industry codes to those at sector-level.
3. For each firm-level industry code match the corresponding digital intensity score using the sector-level data as a reference (look-up table).
4. For each company, calculate revenue-weighted digital intensity score.
5. For each company, classify the revenue-weighted digital intensity score into one of three digital intensity groups (low, mid or high).

#### Step 1: Company revenue per industry code

Depending on the data source, the data might be readily available, or some data processing might be needed. Common data processing requirements include:

• Splitting business segment revenue to multiple industry codes
• Dealing with negative figures
• Dealing with missing revenue breakdown by business segment or industry

Since many companies provide information on their sales per business segment (typically, in annual reports in the notes to the financial statements) it is likely that revenue data is recorded on a per business segment basis, rather than per industry code basis. Nevertheless, business segments can be matched with one or multiple industry codes. This can be done by researchers themselves or such information can be available in the financial databases. In either case, it is common to encounter multiple industry codes assigned to a single business segment. If this is the case, each business segment revenue should be evenly split between industry codes. The justification for such treatment is that typically there is not enough information to assign different weights to individual industry codes. Equal weights reflect equal treatment of all industry codes assigned to a single business segment.

Another data issue, which is sometimes encountered, is negative revenue reported as eliminations of inter-segment sales within a company. We recommend dropping the records with negative revenue, since revenue from each business segment excluding eliminations should sufficiently well reflect the level of company engagement in different industries.

Finally, some companies do not report revenue breakdown by segment and, thus, it might not be possible to get data on revenue per industry code for such companies. The proposed method requires at least one industry code, which is available on a company-level. Such industry code is generally available for any registered company in the form of primary industry code. In some cases, several industry codes might also be available on a firm-level. In either case, the treatment of these industry codes is equivalent to the base case situation, where revenue per business segment is available. The only difference is that instead of using revenue per business segment to allocate revenue per industry code, it is the total revenue of a company, which is used. Primary and secondary industry codes are available in multiple financial databases.

#### Step 2: Converting firm-level industry codes to sector-level codes using concordance tables

This step can be skipped, if both firm-level and sector-level industry codes are expressed using the same industry classification system and the same revision of that system. In other cases, there is a need to harmonize the industry codes on both levels. This is achieved with concordance tables. Once industry codes on firm- and sector-level are matched it is possible to map sector-level digital intensity scores to firm-level in the next step.

Concordance tables can be downloaded from websites of, for example, U.S. Census Bureau [16] or Eurostat [17]. The latter source refers to concordance tables as correspondence tables.

Since it is possible that some industry codes in a concordance table are mapped to more than one code in another system or revision of industry classification, our method requires adjustment of some of the company revenue per industry code figures, which were calculated in the previous step. In line with the logic regarding splitting segment revenue to industry codes, which was presented earlier, we propose the same treatment for cases where concordance tables map a single industry code to multiple codes in another industry classification system. This means that if the concordance table applied maps one industry code to many, our method evenly splits company revenue related to that industry code and allocates that value to the resulting industry codes in another classification system or revision.

While the application of concordance tables, later revenue splitting and allocation of revenues to industry codes should be a straightforward procedure, there is one potential data issue, which reveals itself at this stage. In case the source industry codes are not all from the same revision of an industry classification system, it is possible that the concordance table applied does not map some of the source industry codes to any target industry code. This data issue can be resolved in two ways. Either (1) another concordance table can be used or (2) the same concordance table as previously can be used with both source and target industry codes escalated by one level in the industry classification taxonomy.

We recommend using the first approach, if concordance tables for other revisions of the source industry codes are available. This step can be repeated iteratively until all missing values are replaced with the corresponding target industry codes. Alternatively, and preferably after applying multiple concordance tables, the remaining missing values can be replaced with target industry codes by using the second approach proposed.

In the second approach, the original concordance table is modified by dropping the last digit in the industry codes (both source and target). Also, the firm-level industry codes need to be generalized in the same way. At this point it is important to recognize that dropping the last digit in the industry codes might result in some firm-level records appearing as duplicates. These duplicates appear due to some firm-level records differing between each other only with the last digit of the industry code. If such duplicates appear, they should be merged by summing the revenue figure for all records that are duplicates of each other and removing all, but one. Once this is completed the more generalized concordance table can be reapplied to the more generalized firm-level industry codes. This approach can be iteratively applied until all missing values are replaced with target industry codes.

#### Step 3: Mapping firm-level industry codes to sector-level digital intensity scores

Given that both firm- and sector-level industry codes are expressed using the same industry classification system and its revision, mapping digital intensity scores, which are at sector-level, to industry codes on a firm-level is a matter of using a simple look-up table logic. There should be no data issues present at this stage. However, it is important to validate that there are no missing values, which could result from incomplete industry code coverage of the sector-level digital intensity scores.

#### Step 4: Firm-level revenue-weighted digital intensity score

Once sector digital intensity scores, $SDI_{i}$, are available at firm-level for each industry code, i, the final digital intensity score, $DI_{X}$, for company X is calculated as a weighted average of sector digital intensity scores $SDI_{i}$ (Eq. 1), where weights, $R{\%}_{i,X}^{*}$, are expressed as share of company X revenue coming from industry i. Star in $R{\%}_{i,X}^{*}$ denotes that the revenue share is for the industry code i, which is expressed in the same industry classification system and revision of that system as that of the sector digital intensity score $SDI_{i}$.

(1) $$DI_{X}=\sum_{i=1}^{N}SDI_{i}*R{\%}_{i,X}^{*}$$

#### Step 5: Classification of digital intensity scores into three groups

The final step is classification of firm-level revenue-weighted digital intensity scores into low, medium, and high digital intensity groups. This step is important because of two reasons. First, since the proposed method disaggregates sector level generalizations to firm level, it is an imperative to recognize that the assigned firm-level digital intensity scores cannot be considered as precise figures. Calvino and colleagues [6] report high level of within-sector heterogeneity for many of the digital intensity indicators they consider. Furthermore, they highlight that there can be many alternative ways to aggregate digital intensity indicators into a “global” indicator. This methodological ambiguity reflects the complexity of the underlying phenomenon. Given that digitalization itself is multifaceted, complex, and evolving we do not expect that a single method can fully capture that phenomenon. Second, the proposed method is intended for use with both ordinal and ratio sector-level digital intensity score scales. The lower information content in ordinal scales creates the requirement for simplification of the final method outputs. Overall, given the two reasons discussed above, we consider that the proposed method strikes the right balance between providing useful granularity and acceptable risk of misclassifying companies.

(2) $$g\left(DI_{m}\right)=\;\left\{ \begin{array}{c} Low,\;if\;DI_{m}\leq \;Q_{\frac{1}{3}}\\ Mid,\;if\;DI_{m}>\;Q_{\frac{1}{3}}\;\wedge \;DI_{m}\leq \;Q_{\frac{2}{3}}\\ High,\;otherwise \end{array}\right.$$

The classification of firm-level digital intensity scores, $DI_{m}$, into groups is carried out using a classification function g(x), where $Q_{r}$ is the quantile of reference sector-level digital intensity scores for probability r. The cut-off values between the groups are calculated from the reference data rather than from the firm-level digital intensity scores calculated in Step 4, because there is no guarantee that the sample of companies under analysis is representative of the whole economy. Reference data, on the other hand, is more likely to meet this requirement.

### Method validation

#### Firm-level data

We apply the proposed method to estimate digital intensity scores for two samples of companies. Both selected samples include 1000 largest companies (based on market capitalization), as of 31^st^ August 2020 and based on country of headquarters:

• US Sample: Companies headquartered in the U.S.
• Non-US Sample: Companies headquartered in Australia, Austria, Denmark, Finland, France, Italy, Japan, the Netherlands, Norway, Sweden, and the United Kingdom.

We retrieved the firm-level data from Thomson Reuters Eikon database. For each sample, the distribution of company count by two-digit NAICS code is presented in Fig. 8. We used Eikon Screener App to find unique identifiers (RICs) of publicly listed companies based on respective country of headquarters and market capitalization denominated in USD. Furthermore, we excluded all ETFs (Exchange Traded Funds) and closed-end funds from the sample. We then used Thomson Reuters MS Excel Add-In to retrieve for each company the following items:

• company name
• primary industry code (North American Industry Classification, NAICS)
• primary industry name
• segment code (NAICS)
• segment name
• business total revenue by segment

The samples of companies used in this section were selected for illustrative purposes only. The use of the method is not restricted to countries included in this analysis nor to large companies only. As discussed in the Input data section of this paper, it is the choice of the reference data that determines suitability of the proposed method for the specific sample of companies under investigation. We discuss reference data used in this analysis in the following section.

**Fig. 4:**
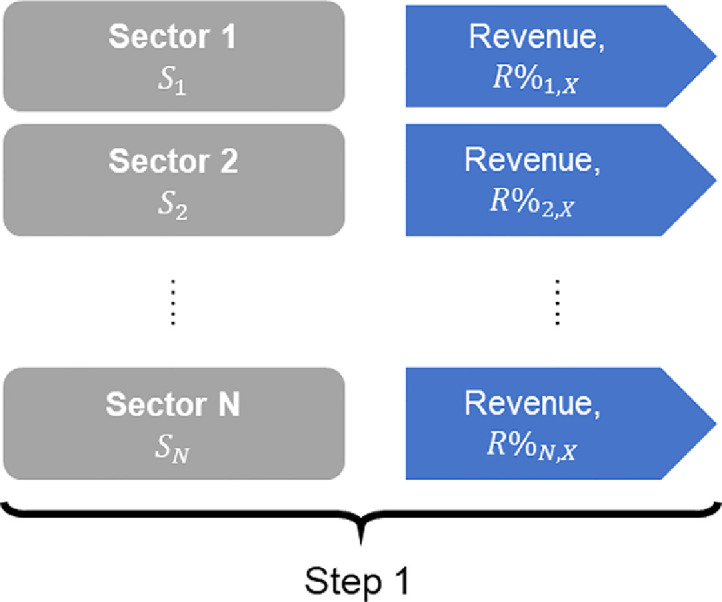
Step 1: For each company, retrieval or calculation of revenue stream broken down by sector.

**Fig. 5:**
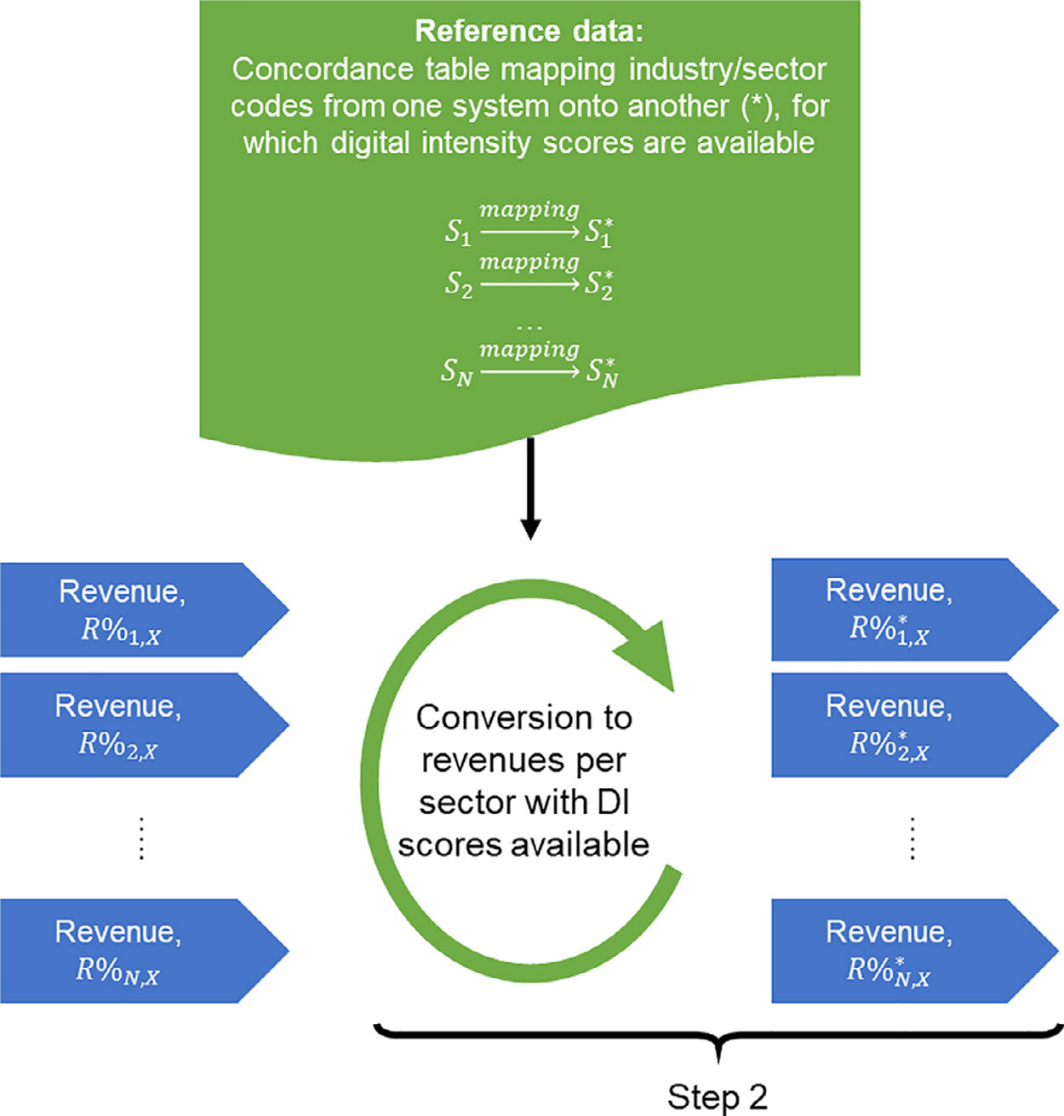
Step 2: Conversion of industry codes related to firm-level revenue streams into another industry classification system, for which sector-level digital intensity scores are available. This step is required only if the firm-level data and sector-level reference data are expressed using a different industry classification systems.

**Fig. 6:**
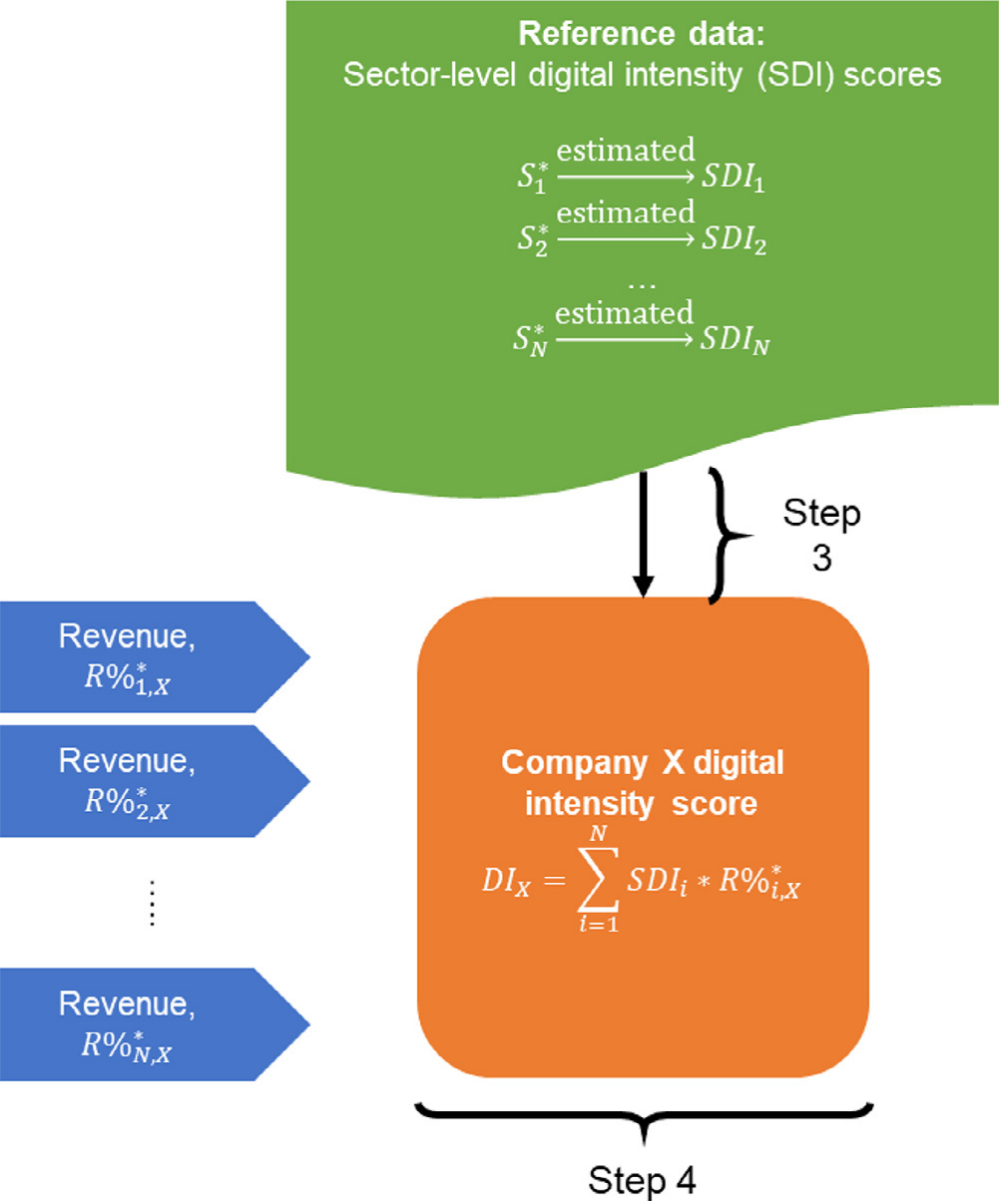
Step 3: Matching of revenue streams and their corresponding industry codes with sector-level digital intensity scores, which come from reference data. Step 4: Calculation of revenue-weighted digital intensity score for each company.

**Fig. 7:**
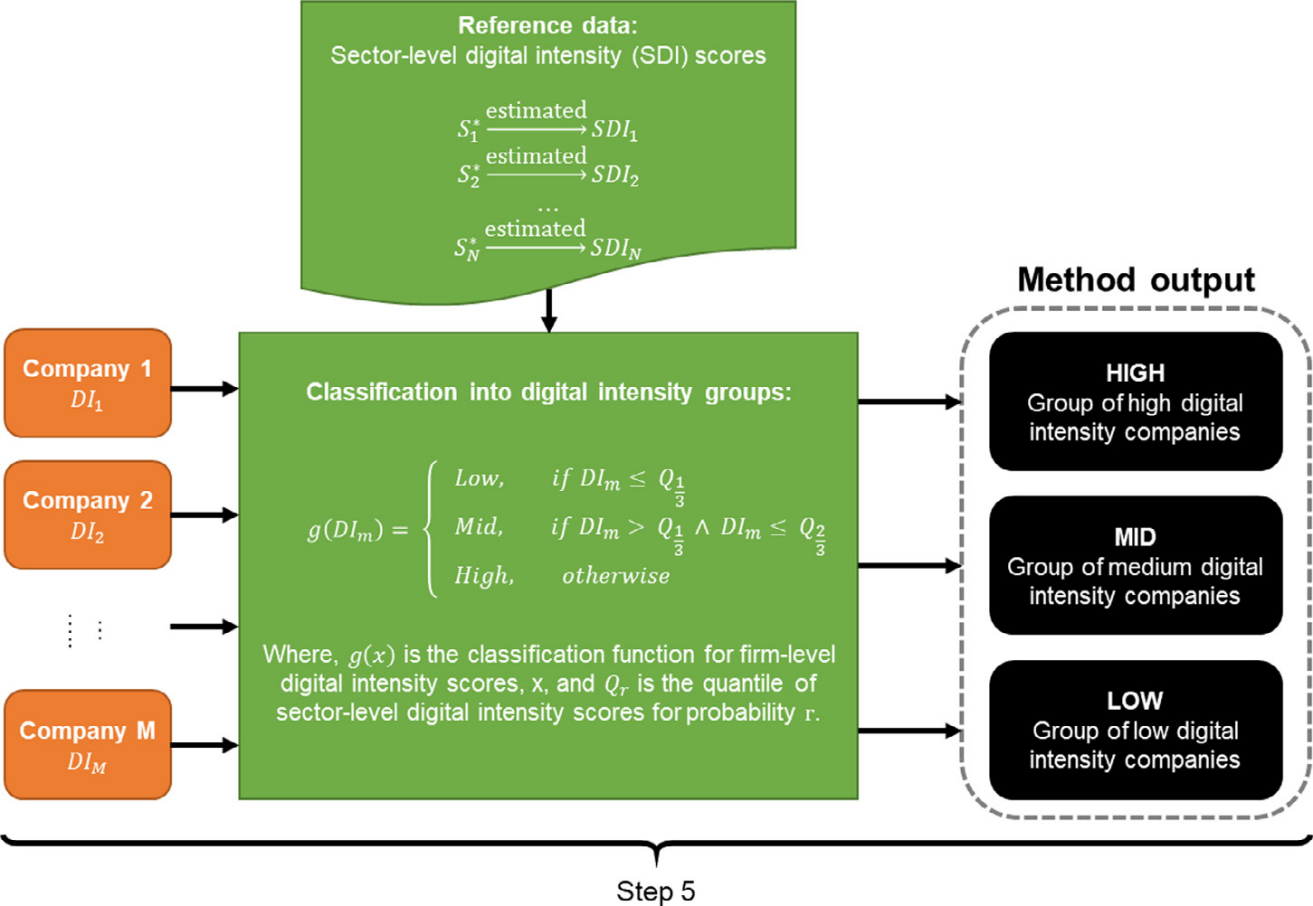
Step 5: Classification of firms into digital intensity groups based on firm-level digital intensity scores and using cut-off points (quantiles with probabilities 1/3 and 2/3) based on reference sector-level digital intensity scores.

**Fig. 8:**
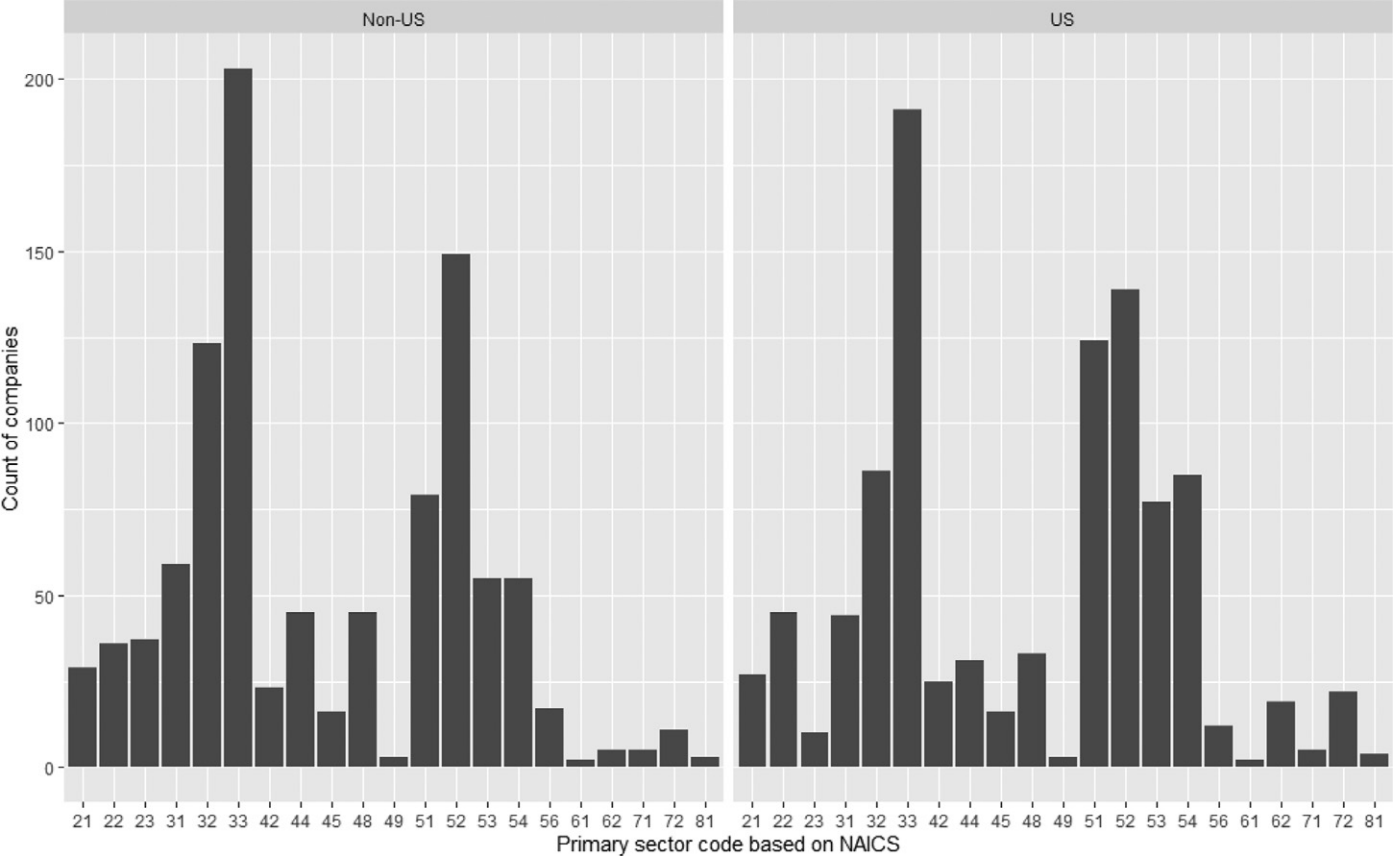
Count of companies by sector (based on first two digits of primary NAICS code)

Fig. 4, Fig. 5, Fig. 6, Fig. 7, Fig. 8.

#### Reference data

Analysis of our samples required two types of reference data, which were concordance tables and sector-level digital intensity scores. Since industry codes available in the firm-level data (NAICS codes) and sector-level data (ISIC codes) were expressed using different classification systems, we needed to employ concordance tables to translate between them. We relied on concordance tables mapping NAICS codes to ISIC codes available from U.S. Census Bureau [16]. Furthermore, since some NAICS codes were expressed using revisions of NAICS classification other than the latest, 2017 revision, in some cases we needed to map these older NAICS to more recent revisions of NAICS. This mapping was also done using concordance tables available from the same source. Sector-level digital intensity scores are discussed in more detail in the remainder of this section.

The 12 countries, which are covered by the sample, were selected, because they are included in the OECD taxonomy of digital intensive industries [6], which is the source of our reference data covering sector-level digital intensity scores. We consider that this reference data is a good example of input that is suitable for the proposed method. In case of OECD taxonomy, digitalization is considered through multiple indicators, thus capturing numerous facets of this complex phenomenon. Other alternative sector-level digital intensity scores, such as those calculated by Brynjolfsson and colleagues [11], could be used as well, although alignment of the selected samples and the reference data would not be as good due to differences in geographic coverage. Users of the proposed method must decide which reference data for sector-level digital intensity is suitable for their research question and design.

Despite the fact that Calvino and colleagues [6] do not report sector-level digital intensity scores directly in their paper, we can replicate their ultimate “global” taxonomy results for all, but one sector, thus achieve 97.22% agreement between our results. Based on our calculation of “global” sector-level digital intensity scores “Transport equipment” sector falls into one digital intensity group lower than what is presented in the results of Calvino and colleagues [6]. We attribute the difference in our replication results to the fact that our classification of sectors into groups of “global” indicator for digital intensity relies on indicator-level digital intensity scores aggregated across countries and years (this data is openly available from OECD via a StatLink dx.doi.org/10.1787/888933617434). Thus, variability on country- or year-level could lead to different classification of “Transport equipment” sector. Nevertheless, we consider that the high degree of alignment between our results is sufficient to rely on our estimation of sector-level digital intensity scores in the reminder of the analysis. The sector-level digital intensity scores used in this analysis are presented in Table 1 and are also available for download from the supplementary materials available with this article.

**Table 1:** Reference data for sector-level digital intensity scores.

Sector	ISIC code (rev. 4)	Digital Intensity Score*
Agriculture, forestry, fishing	01–03	0.0463
Mining and quarrying	05–09	0.2361
Food products, beverages and tobacco	10–12	0.3254
Textiles, wearing apparel, leather	13–15	0.4246
Wood and paper products, and printing	16–18	0.4563
Coke and refined petroleum products	19	0.3532
Chemicals and chemical products	20	0.4087
Pharmaceutical products	21	0.3651
Rubber and plastics products	22–23	0.4365
Basic metals and fabricated metal products	24–25	0.3690
Computer, electronic and optical products	26	0.5648
Electrical equipment	27	0.5185
Machinery and equipment n.e.c.	28	0.5324
Transport equipment	29–30	0.6157
Furniture; other manufacturing; repairs of computers	31–33	0.5754
Electricity, gas, steam and air cond.	35	0.3016
Water supply; sewerage, waste management	36–39	0.3016
Construction	41–43	0.2698
Wholesale and retail trade, repair	45–47	0.5926
Transportation and storage	49–53	0.3194
Accommodation and food service activities	55–56	0.2870
Publishing, audiovisual and broadcasting	58–60	0.6157
Telecommunications	61	0.8796
IT and other information services	62–63	0.8241
Finance and insurance	64–66	0.8222
Real estate	68	0.0741
Legal and accounting activities, etc.	69–71	0.6620
Scientific research and development	72	0.6204
Advertising and market research; other business services	73–75	0.6806
Administrative and support service activities	77–82	0.6528
Public administration and defence	84	0.5333
Education	85	0.3944
Human health activities	86	0.4333
Residential care and social work activities	87–88	0.4111
Arts, entertainment and recreation	90–93	0.4889
Other service activities	94–96	0.6167

⁎ These scores were estimated following the methodology developed by Calvino and colleagues [6] and using data available from OECD via a StatLink dx.doi.org/10.1787/888933617434. The scores themselves do not have direct interpretation other than providing ranking of sectors in terms of their digital intensity.

These sector-level digital intensity scores are used in the analysis as a reference look-up table for assigning digital intensity scores to company-level streams of revenue coming from activity in different sectors. Once revenue-weighted digital intensity scores are calculated for each company, we use again the reference look-up table to compare these scores against cut-off points between low, medium and high digital intensity sectors. These cut-off points are quantiles in the reference look-up table digital intensity scores corresponding to 1/3 and 2/3 probabilities. Thus, given our reference data, firms with revenue-weighted digital intensity score below 0.386 are classified as low digital intensity, those with scores above 0.568 are classified as high digital intensity, and those in between are medium digital intensity.

#### Efficiency of the method

**Fig. 9:**
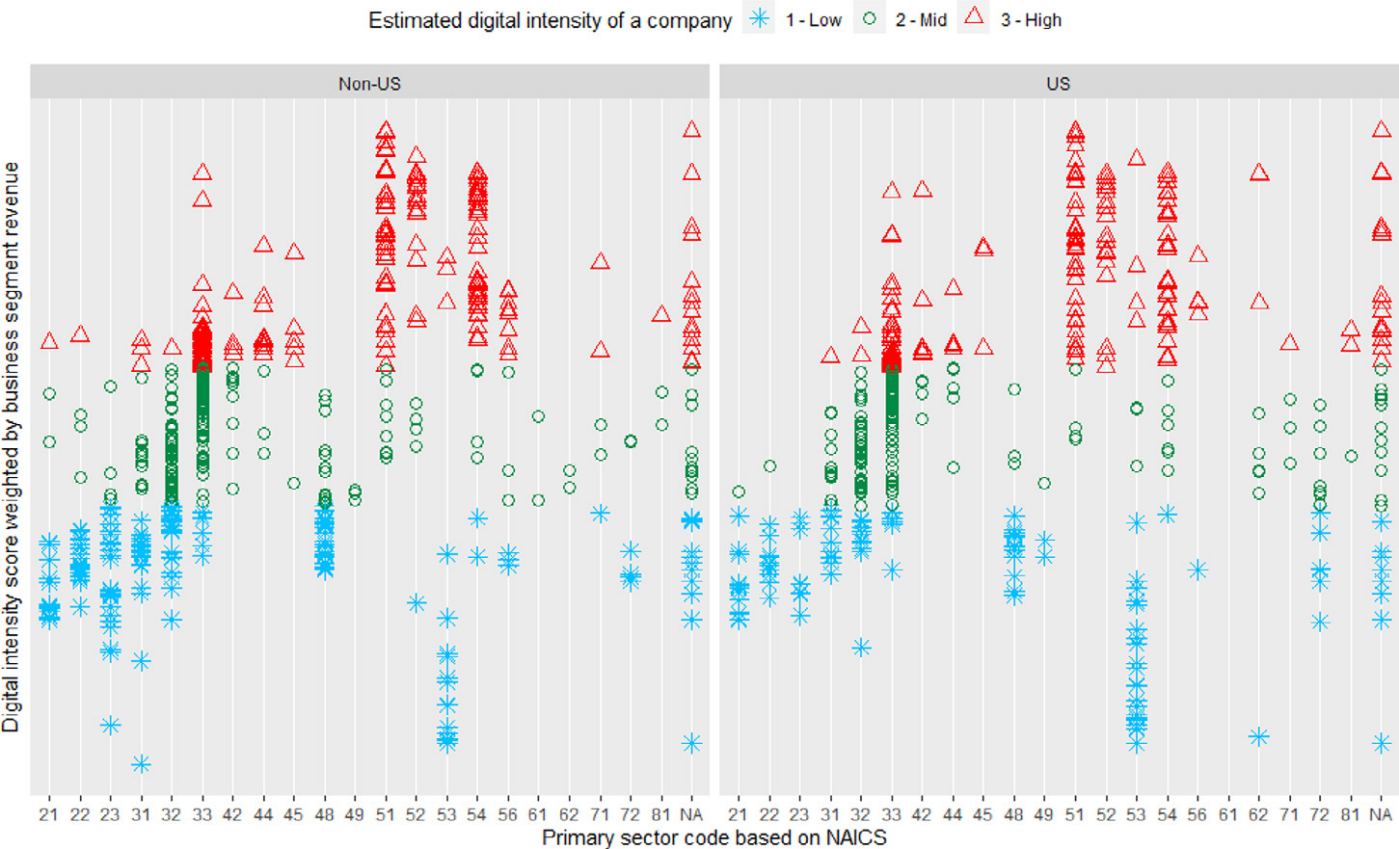
Visual representation of method output for the two data samples.

Using the input data and following the proposed method (steps 1-5) yields a classification of firm-level digital intensity into three groups as presented in Fig. 9.

In the absence of any other information, the input data was enough to estimate digital intensity for the sample companies on a firm-level, thus demonstrating the efficiency of the proposed method, given suitable sector-level reference data is available. Relatively low data requirement and accessibility of the required data make the proposed method practically feasible for use. Such data efficiency is the primary advantage of the proposed method, which despite the lack of more detailed data on company digitalization can be used in a wide range of research work.

Another aspect of the proposed method is procedural clarity, which leads to higher replicability and comparability in the studies investigating or utilizing firm-level digitalization measures. Not only should the description of the proposed method provided in this paper be used to inform researchers regarding the method steps, but also R code included in the supplementary material should provide means for higher replicability.

Finally, given the automation of data processing using R script and separation of the method inputs into firm-level data and reference data, this method provides means for research updatability. Once new firm-level data or reference data on sector-level digital intensity becomes available, the requirement for resources needed to recalculate and update results is low.

#### Comparison of firm digital intensity based on primary industry only and segment level industries

As we noted in the description of Step 1 of the proposed method, primary industry codes can be used to supplement the firm-level data in cases where revenue breakdown by business segment is not available for some companies. However, it is important to point out that there is a potential trade-off related to inclusion of companies with lacking data on segment revenue. While it is likely that researchers applying the proposed method will not have full coverage of firm-level business segment revenue data for their samples, we would recommend using the proposed method only in cases where majority of the sample has such data available.

To demonstrate the difference in the results, which are based on data with full access to business segment revenue and data with primary industry codes only, we provide comparative results in this section.

We used the input data consisting of the same two samples as in the previous section as the starting point for this analysis. After excluding companies, which did not have revenue breakdown by business segment, were left with 678 and 786 observations for US and Non-US samples, respectively.

**Fig. 10:**
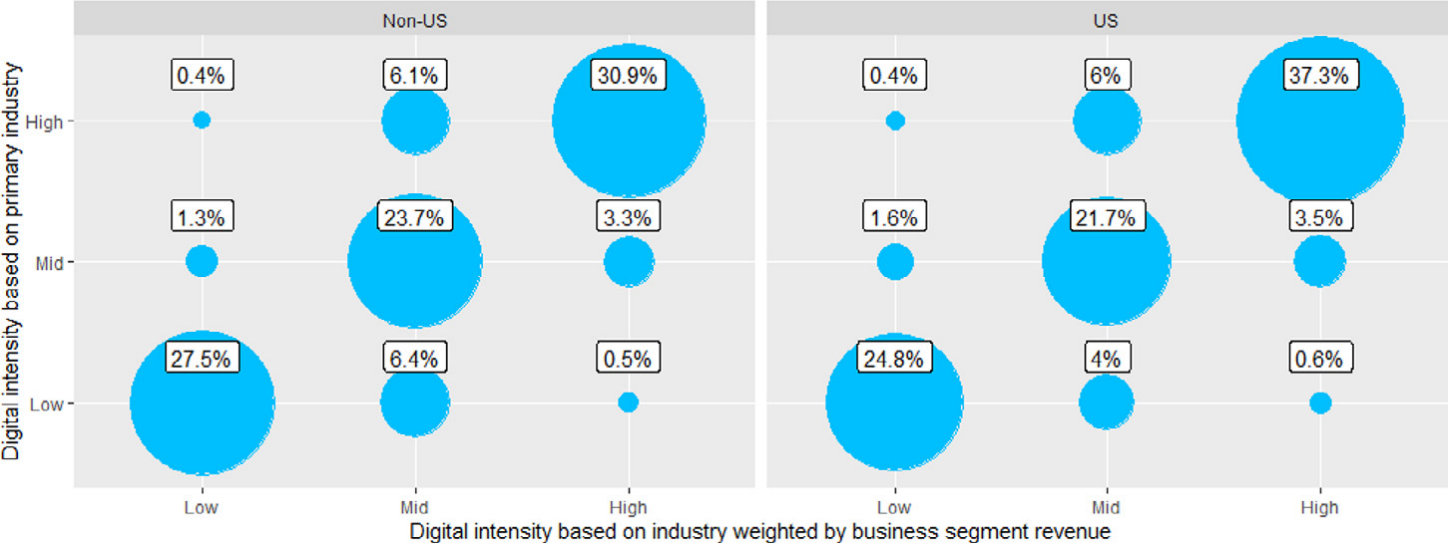
Comparison of method results with and without firm-level business segment revenue data.

Using these restricted samples, we recalculated the results of the proposed method. We refer to these results as “digital intensity based on industry weighted by business segment revenue”. Thereafter, we removed business segment revenue information from the restricted samples and recalculated the results. Since this second application of the proposed method could not use business segment revenue as weights to calculate firm-level digital intensity, only information regarding primary industry of each company was used. We refer to these results as “digital intensity based on primary industry”. Comparison of the results from both runs is presented in Fig. 10.

There is an overall alignment between the results from each calculation run, as presented in Table 2. Cohen's kappa for both samples is relatively high, thus indicating agreement between the two approaches. However, this result is expected, as the null hypothesis for Cohen's kappa is random grouping of the observations. In our case, we are more interested to detect if there is difference between the two approaches in terms of groupings. While simple percentage agreement is above 80% for both samples, the permutation test rejects, at 5% significance level, the hypothesis that the agreement is 100%. Thus, we conclude that there is higher information content in the approach relying on business segment revenue figures and the resulting revenue-weighted digital intensity scores. Our recommendation is to use to the extent possible firm-level data with information on revenue per industry or business segment. In our view, this is a superior approach to one relying solely on primary industry codes.

**Table 2:** Agreement in classification of companies into digital intensity groups between results with and without firm-level business segment revenue data.

Sample	Non-US	US
Observation count	786	678
Cohen's kappa	0.731	0.752
Simple percentage agreement	82.1% *(79.3%, 84.7%)*†	83.8% *(81.0%, 86.4%)*†

† Values in parenthesis show estimated confidence interval for α = 5% using permutation test with 5000 bootstraps.

#### Conclusion and limitations

Overall, the proposed method exhibits the key intended property, which is efficient estimation of firm-level digital intensity, while utilizing data that is readily available for large samples of companies. By leveraging information on the level of business activity of companies in different industries and sectors the proposed method allows scholars to tap into results from previous research on digital intensity of sectors. The results from validation of the method against two samples of companies with 1000 observations each reveal that classification of firms into low, medium and high digital intensity groups is significantly different from alternative classification, where only information on firm primary industry is used. Thus, we conclude that the proposed method using revenue-weighted digital intensity scores produces superior estimates of firm digital intensity.

Since the proposed method relies on sector-level reference data on digitalization, its results can be only as good as the quality of the reference data. While this presents a limitation, it provides also a benefit in the form of updatability of the research results. Simply swapping the reference data to a different or newer version, with no further alternations in the estimation procedure, generates potentially more appropriate or more up-to-date results. This means that the proposed method is flexible in the sense that researchers can choose reference data to match the geography, timeframe and other parameters of their firm-level data. Furthermore, even if firm-level data on digitalization is available to some extent, for example covering only certain aspects of digitalization, the proposed method can be used to augment or supplement the data, thus potentially providing better operationalization of firm digitalization.

Finally, the proposed method is intended to increase transparency and replicability of research on digitalization. The supplementary material included with this paper comprises of not only input data used in the method validation section, but also source code (in R language), which allows for exact reproduction of the results. Thanks to the source code and relative availability of input data, which is suitable for the proposed method, large samples of companies can be classified into digital intensity groups in a manner, which is transparent to the research community.

The proposed method can also be further developed to incorporate other measures of firm engagement in different sectors. For example, apart from relying on revenue as an indicator of sector engagement, sourcing relationships could also provide useful input to the method. Analysis of sourcing relationships allow for derivation of value-add distribution across supply chain [18], [19], [20] and thus could provide an up-stream perspective on digitalization.

## Declaration of Competing Interest

The authors declare that they have no known competing financial interests or personal relationships that could have appeared to influence the work reported in this paper.

## Appendix Supplementary materials

Image, application 1

Image, application 2

Image, application 3

Image, application 4

Image, application 5

Image, application 6

Image, application 7

Image, application 8
