# Supplementary material for: Estimating firm digitalization: A method for disaggregating sector-level digital intensity to firm-level
Source: MethodsX. 2021 Jan 18;8:101233. doi: 10.1016/j.mex.2021.101233 (PMC8374197; doi:10.1016/j.mex.2021.101233)
Supplement: Supplementary file 1 [file mmc1.docx]

**Supplementary material:**

- R code
- Report generated from R, where all the calculation steps and outputs are visible
- Sample data:
  - Revenue breakdown by business segment for 2000 sample companies, primary and business segment industry codes
  - Reference data with sector level digital intensity scores and taxonomy
  - Concordance tables mapping different industry classification systems and revisions to each other
  - Sector-level digital intensity scores calculated based on OECD data available from dx.doi.org/10.1787/888933617434
