# Supplementary material for: Estimating firm digitalization: A method for disaggregating sector-level digital intensity to firm-level
Source: MethodsX. 2021 Jan 18;8:101233. doi: 10.1016/j.mex.2021.101233 (PMC8374197; doi:10.1016/j.mex.2021.101233)
Supplement: Supplementary file 7 [file mmc7.pdf]

Supplementary material to “Estimating firm digitalization: A method for disaggregating sector-level digital intensity to firm-level”: Report generated from execution of R code

2020-10-15

```
###########################
# Load libraries
###########################
# reading Excel files into R
library(readxl)
library(readr)

# tool for reshaping data
library(reshape2)

# for plotting diagrams
library("ggplot2")
library(scales)
```

```
##
## Attaching package: 'scales'

## The following object is masked from 'package:readr':
##
##     col_factor
```

```
library(gridExtra)
library(tidyquant)
```

```
## Loading required package: lubridate

##
## Attaching package: 'lubridate'

## The following object is masked from 'package:base':
##
##     date

## Loading required package: PerformanceAnalytics

## Loading required package: xts

## Loading required package: zoo

##
## Attaching package: 'zoo'

## The following objects are masked from 'package:base':
##
##     as.Date, as.Date.numeric

##
## Attaching package: 'PerformanceAnalytics'
```

```
## The following object is masked from 'package:graphics':
##
##     legend

## Loading required package: quantmod

## Loading required package: TTR

## Registered S3 method overwritten by 'quantmod':
##   method            from
##   as.zoo.data.frame zoo

## Version 0.4-0 included new data defaults. See ?getSymbols.

## == Need to Learn tidyquant? ===========================================================
==================================================================================
## Business Science offers a 1-hour course - Learning Lab #9: Performance Analysis & Port
folio Optimization with tidyquant!
## </> Learn more at: https://university.business-science.io/p/learning-labs-pro </>

# string manipulation
library(stringr)

# will be used to create lagged variable
library(DataCombine)

# data manipulation, sorting and grouping
library(dplyr)

##
## Attaching package: 'dplyr'

## The following objects are masked from 'package:xts':
##
##     first, last

## The following objects are masked from 'package:lubridate':
##
##     intersect, setdiff, union

## The following object is masked from 'package:gridExtra':
##
##     combine

## The following objects are masked from 'package:stats':
##
##     filter, lag

## The following objects are masked from 'package:base':
##
##     intersect, setdiff, setequal, union

library(data.table)

##
## Attaching package: 'data.table'
```

```
## The following objects are masked from 'package:dplyr':
##
##     between, first, last

## The following object is masked from 'package:DataCombine':
##
##     shift

## The following objects are masked from 'package:xts':
##
##     first, last

## The following objects are masked from 'package:lubridate':
##
##     hour, isoweek, mday, minute, month, quarter, second, wday, week,
##     yday, year

## The following objects are masked from 'package:reshape2':
##
##     dcast, melt

library(purrr)

##
## Attaching package: 'purrr'

## The following object is masked from 'package:data.table':
##
##     transpose

## The following object is masked from 'package:scales':
##
##     discard

library(tidyr)

##
## Attaching package: 'tidyr'

## The following object is masked from 'package:reshape2':
##
##     smiths

# Load library for inter-rater reliability analysis
library(irr)

## Loading required package: lpSolve

# Load library for bootstrapping
library(bootstrap)

###########################
# Load data

# write.table(InputData, file = "temp.csv")
# length(unique(InputData$`Company Name`))
###########################
# Set working directory
```

```
setwd("C:/Users/k80278/OneDrive - Aalto University/MethodsX 2020/Additional documents")

#%%%%%%%%%%%%%%%%%%%%%%%%%%%%%%%%%%%%%%%%%%%%%%%%%%%%%%%%%%%%%%%%%%%%%%%%%%%%%%%%
#%%%%%%%%%%%%%%%%%% Step 1 of the method %%%%%%%%%%%%%%%%%%%%%%%%%%%%%%%%%%%%%%%%%
#%%%%%%%%%%%%%%%%%%%%%%%%%%%%%%%%%%%%%%%%%%%%%%%%%%%%%%%%%%%%%%%%%%%%%%%%%%%%%%%%
# Load data with two samples of companies and their segment and priary NAICS codes.
# Warnings about NULL values relate to missing data on segment level for some companies.
  USData <- read_excel("US Sample.xlsx",
                       col_types = c("text", "text", "text", "text", "numeric", "text"),
                       na = "NULL")

## Warning in read_fun(path = enc2native(normalizePath(path)), sheet_i = sheet, :
## Expecting numeric in E4447 / R4447C5: got 'Unable to collect data for the field
## 'TR.BGS.BusTotalRevenue' and some specific identifier(s).'

## Warning in read_fun(path = enc2native(normalizePath(path)), sheet_i = sheet, :
## Expecting numeric in E4448 / R4448C5: got 'Unable to collect data for the field
## 'TR.BGS.BusTotalRevenue' and some specific identifier(s).'

  # Respond to warnings, which might impact later data processing
  # Manually assign "NA" to cells which include message "Unable to collect data..." gener
ated by the original database
USData$`Segment Name`[which(substring(USData$`Segment Code`,1,6) == "Unable")] = NA
USData$`Segment Code`[which(substring(USData$`Segment Code`,1,6) == "Unable")] = NA

NonUSData <- read_excel("Non-US Sample.xlsx",
                        col_types = c("text", "text", "text", "text", "numeric", "text"),
                        na = "NULL")

# Get a mapping table from 2017_NAICS to ISIC_4
# Source: https://www.census.gov/eos/www/naics/concordances/2017_NAICS_to_ISIC_4.xlsx
# Original file converted to CSV before import, otherwise leading zeros are lost
Naics2017ToIsic4 <- read_csv("2017_NAICS_to_ISIC_4.csv",
                             col_types = cols(`2017\nNAICS\nUS` = col_character()))

# Get a mapping table from 2012_NAICS to ISIC_4
# Source: https://www.census.gov/eos/www/naics/concordances/2012%20NAICS_to_ISIC_4.xlsx
# Original file converted to CSV before import, otherwise leading zeros are lost
Naics2012ToIsic4 <- read_csv("2012 NAICS_to_ISIC_4.csv",
                             col_types = cols(`2012\nNAICS\nUS` = col_character(),
                                              X6 = col_skip()))

## Warning: Missing column names filled in: 'X6' [6]

# Get a mapping table from 2007_NAICS to ISIC_4
# Source: https://www.census.gov/eos/www/naics/concordances/2007_NAICS_to_ISIC_4.xls
# Original file converted to CSV before import, otherwise leading zeros are lost
Naics2007ToIsic4 <- read_csv("2007_NAICS_to_ISIC_4.csv",
                             col_types = cols(`2007 NAICS US` = col_character(),
                                              X6 = col_skip()))

## Warning: Missing column names filled in: 'X6' [6]

# Get a mapping table from 1997_NAICS_to_2002_NAICS
# This is needed, because some NAICS codes in the data were assigned very long time ago
# Source: https://www.census.gov/eos/www/naics/concordances/1997_NAICS_to_2002_NAICS.xls
Naics1997To2002 <- read_csv("1997_NAICS_to_2002_NAICS.csv",
```

```
                      col_types = cols(`EXPLANATION OF PARTS` = col_skip(),
                                       NAICS02 = col_character(), NAICS97 = col_character
()))

# Get a mapping table from 2002_NAICS_to_2007_NAICS
# This is needed, because some NAICS codes in the data were assigned very long time ago
# Source: https://www.census.gov/eos/www/naics/concordances/2002_to_2007_NAICS.xls
Naics2002To2007 <- read_csv("2002_to_2007_NAICS.csv",
                            col_types = cols(`2002 NAICS Code` = col_character(),
                                             `2007 NAICS Code` = col_character()),
                            skip = 2)

# Get an OECD "Taxonomy of sectors by quartile of digital intensity, 2013-15" file.
# Original source is http://dx.doi.org/10.1787/888933617434 - the file is unaltered.
# All underlying indicators are expressed as sectoral intensities. For each
# indicator, the sectoral values are averages across countries and years. These
# values are then standardised relative to the mean, such that the resulting
# series by indicator have mean zero and standard deviation 1.
OecdDigitalIntensity <- read_excel("OECD-Digital intensity.xlsx",
                                   col_types = c("text", "numeric", "numeric",
                                                 "numeric", "numeric", "numeric",
                                                 "numeric", "numeric", rep("skip", 16))
, skip = 12)

## New names:
## * `` -> ...1

# Drop rows, which don't contain sectoral intensities
OecdDigitalIntensity <- OecdDigitalIntensity[1:36, ]

# Get a table with sectoral taxonomy of digital intensity: "global" indicator.
# That is Table 3 from F. Calvino, C. Criscuolo, L. Marcolin, and M.
# Squicciarini, "A taxonomy of digital intensive sectors," 2018, [Online].
# Available: https://www.oecd-ilibrary.org/docserver/f404736a-en.pdf).
OecdGlobalTaxonomy <-read_excel(
  "Table 3 - Sectoral taxonomy of digital intensity (Calvino et al., 2018).xlsx")

##########################################################
# The first step in data processing is the calculation of Digital Intensity
# scores on sector level.
##########################################################

# Add name to the first column with sector names
colnames(OecdDigitalIntensity)[1] <- c("Sector")

# Based on the methodology proposed by F. Calvino, C. Criscuolo, L. Marcolin,
# and M. Squicciarini, "A taxonomy of digital intensive sectors," (2018), we
# calculate a Digital Intensity score of a sector as a weighted ranking of each
# indicator. The weights are 1/36, which is the number of sectors, and missing
# values are omitted when calculating the average. However, we do not have
# access to country- and year- level data, thus we are working with averages.
# While this might result in minor differences in our results of sectoral
# classification, it should not meaningfully impact the overall classification.

# Create a dataframe to store the ranking
```

```
OecdRank <- as.data.frame(apply(OecdDigitalIntensity[, 2:8],2,rank,na.last = TRUE, ties.m
ethod = "min"))

# Since NAs are assigned rankings here, they need to be replaced with NAs again
OecdRank = OecdRank*!is.na(OecdDigitalIntensity[, 2:8])
OecdRank[OecdRank == 0] <- NA

# These rankings can be weighted now with 1/36 weight
OecdRank <- OecdRank/36

# The "global" score for each sector is the average of the weighted rankings
# (ignoring NAs)
OecdRank$DiScore <- apply(OecdRank, 1, function(x) sum(x,na.rm = TRUE)/sum(!is.na(x)))

# Add sector names
OecdRank$Sector <- OecdDigitalIntensity$Sector

# These "global" scores can be compared now with the final classification of
# sectors into quartiles, which was presented in by Calvino and colleagues
# (2018). To do the comparison, we first need to assign our scores into
# quartiles and, then, compare these.
OecdRank$Quartile <- 1
OecdRank$Quartile[OecdRank$DiScore > quantile(OecdRank$DiScore, 0.25) &
                    OecdRank$DiScore <= quantile(OecdRank$DiScore, 0.5)] <- 2
OecdRank$Quartile[OecdRank$DiScore > quantile(OecdRank$DiScore, 0.5) &
                    OecdRank$DiScore <= quantile(OecdRank$DiScore, 0.75)] <- 3
OecdRank$Quartile[OecdRank$DiScore > quantile(OecdRank$DiScore, 0.75)] <- 4

# Convert text into numeric quantile in OECD "global" taxonomy
OecdGlobalTaxonomy$OecdQuartile <- 1
OecdGlobalTaxonomy$OecdQuartile[OecdGlobalTaxonomy$`Quartile13-15` == "Medium-low"] <- 2
OecdGlobalTaxonomy$OecdQuartile[OecdGlobalTaxonomy$`Quartile13-15` == "Medium-high"] <- 3
OecdGlobalTaxonomy$OecdQuartile[OecdGlobalTaxonomy$`Quartile13-15` == "High"] <- 4

# Since both OecdGlobalTaxonomy and OecdRank have sectors arranged in the same
# order, it is possible to simply bind them together, even if they don't have
# common key. Note, that the naming convention for sector names is different in
# both tables.
QuartileComparison <- bind_cols(OecdRank, OecdGlobalTaxonomy)

# Compare the assignment to quartiles
QuartileComparison$Diff <- abs(QuartileComparison$Quartile - QuartileComparison$OecdQuart
ile)
# Number of sectors with different assignment to quartiles
sum(!QuartileComparison$Diff == 0)

## [1] 1

# Only one sector is classified differently, which we consider a good agreement
# of our results with the reference methodology. The difference is likely to
# arise from outliers on country and/or year level.

##########################################################
# Define functions for data processing
##########################################################
```

```
# Clean the data. For companies where there is segment-level sales and NAICS
# data, drop rows where there is no information with NAICS codes. If on
# segment-level there is no NAICS data, we need to keep these companies and work
# with primary NAICS. Returns data, which is ready for matching with ISIC codes
cleanData<-function(InputData)
{
  # Find rows which don't contain NAICS codes. This primarily indicates the problem with
intercompany revenue eliminations,
  # which is not useful in this analysis.
  # First find those rows and mark them for future deletion in hasNaics column
  InputData$hasNaics <- !is.na(as.numeric(str_sub(InputData$`Segment Code`, 1,1)))

  # However, we want to keep companies, which don't have segment-level details, but are s
till included in the sample
  # Keep those rows, which have segment-level data and move rest to another dataframe
  InputDataNoSegment <- (InputData %>% filter(hasNaics == FALSE))
  InputData <- (InputData %>% filter(hasNaics == TRUE))

  # Assign NA to segment name and code, where there is no segment-level NAICS
  InputDataNoSegment$`Segment Name` = NA
  InputDataNoSegment$`Segment Code` = NA
  InputDataNoSegment$`Business Total Revenues (Calculated)` = NA
  # Then remove duplicates
  InputDataNoSegment <- unique(InputDataNoSegment)
  # Then check if these companies have some segment-level data with NAICS codes availabil
e.
  # That means, check if they are included in the other data frame as well
  InputDataNoSegment$hasSegmentNaics <- InputDataNoSegment$`Company Name` %in% InputData$
`Company Name`
  # Keep only those that were missing from the other table
  InputDataNoSegment <- (InputDataNoSegment %>% filter(hasSegmentNaics == FALSE))
  # Drop the last column with auxiliary information
  InputDataNoSegment <- InputDataNoSegment[,c(-9)]
  # Mark that these companies should be kept, when moved back to the other table
  InputDataNoSegment$hasNaics = TRUE
  # Move these companies back to the other data
  InputData <- dplyr::bind_rows(InputData, InputDataNoSegment)
  # Remove the auxiliary table
  rm(InputDataNoSegment)

  # Then drop rows marked for deletions
  InputData <- (InputData %>% filter(hasNaics == TRUE))
  #Then drop auxiliary column "hasNaics"
  InputData <- InputData[,c(-8)]

  # Shorten the name of primary NAICS
  colnames(InputData)[6] <- c("PrimaryNAICS")

  # Shorten the name of Company Name variable
  colnames(InputData)[1] <- c("Name")

  # For companies, which don't have segment-level NAICS, move primary NAICS as segment NA
ICS
```

```
  # Also assign an arbitrary revenue of 1, as they don't have any information on segment
revenue
  InputData$`Business Total Revenues (Calculated)`[is.na(InputData$`Segment Code`)] = 1
  InputData$`Segment Code`[is.na(InputData$`Segment Code`)] = InputData$PrimaryNAICS[is.n
a(InputData$`Segment Code`)]

  # Split the column with NAICS codes into separate column for each code
  # Based on the manual inspection of data, there are up to 4 codes in one cell
  InputData <- InputData %>% separate("Segment Code", c("c1", "c2", "c3", "c4"), ",")

  # Ensure that all codes are numeric. Replace text codes with NAs.
  InputData$c1[is.na(as.numeric(InputData$c1))] = NA
  InputData$c2[is.na(as.numeric(InputData$c2))] = NA
  InputData$c3[is.na(as.numeric(InputData$c3))] = NA
  InputData$c4[is.na(as.numeric(InputData$c4))] = NA

  # Calculate revenue per NAICS code
  InputData$RevenuePerCode <- InputData$`Business Total Revenues (Calculated)`/
    (as.numeric(!is.na(InputData$c1)) +
       as.numeric(!is.na(InputData$c2)) +
       as.numeric(!is.na(InputData$c3)) +
       as.numeric(!is.na(InputData$c4)))

  # Calculate sum of total revenue for all companies
  # It will be used to check that data manipulation doesn't alter the underlying values
  TotalRevenue <<- sum(InputData$`Business Total Revenues (Calculated)`)

  # Stack columns with Naics code numbers on top of each other (to get one column for cod
e)
  # Melt, that is convert wide form data to long form, while keeping variables,
  # which are fixed as columns
  InputDataMolten <- reshape2::melt(InputData, id.vars = c("RIC", "Name", "Segment Name",
                                                           "Business Total Revenues (Calc
ulated)",
                                                           "PrimaryNAICS", "RevenuePerCod
e", "Sample"))

  # Drop rows with no Naics codes (equal to NA)
  InputDataMolten <- (InputDataMolten %>% filter(!is.na(value)))

  # Drop column called "variable"
  InputDataMolten <- InputDataMolten[,c(-8)]

  # Check how long Naics codes are
  length((InputDataMolten %>% filter(str_length(value) > 6))[,1])
  length((InputDataMolten %>% filter(str_length(value) < 6))[,1])
  length((InputDataMolten %>% filter(str_length(value) < 5))[,1])

  # Create a column showing code length
  InputDataMolten$CodeLengeth <- str_length(InputDataMolten$value)

  # Append "0" to codes that have 5 characters
  InputDataMolten$value[InputDataMolten$CodeLengeth == 5] <-
    str_c(InputDataMolten$value[InputDataMolten$CodeLengeth == 5], "0")
```

```
  # Drop column with code length
  InputDataMolten <- InputDataMolten[,c(-9)]

  # Rename column "value" to SegmentNAICS
  colnames(InputDataMolten)[8] <- c("SegmentNAICS")

  # Clean the working space by reverting back to work with InputData
  InputData <- InputDataMolten
  rm(InputDataMolten)

  # Check if there was any change in total revenue
  TotalRevenue-sum(InputData$RevenuePerCode[!is.na(InputData$RevenuePerCode)])

  # For each company sum revenue by NAICS code
  InputData <- InputData %>%
    group_by(RIC, Name, PrimaryNAICS, SegmentNAICS, Sample) %>%
    summarise(RevenuePerCode = sum(RevenuePerCode))

  # Rename 6 digit codes with Segment NAICS
  colnames(InputData)[4] <- c("NAICS")

  # Check if there was any change in total revenue
  message("Check if there was any change in total revenue")
  message("Difference is ", TotalRevenue-sum(InputData$RevenuePerCode[!is.na(InputData$Re
venuePerCode)]))

  # Return data ready for further processing
  return(InputData)
}

#%%%%%%%%%%%%%%%%%%%%%%%%%%%%%%%%%%%%%%%%%%%%%%%%%%%%%%%%%%%%%%%%%%%%%%%%%%%%%%%%%
#%%%%%%%%%%%%%%%%% Step 2 of the method %%%%%%%%%%%%%%%%%%%%%%%%%%%%%%%%%%%%%%%%%%
#%%%%%%%%%%%%%%%%%%%%%%%%%%%%%%%%%%%%%%%%%%%%%%%%%%%%%%%%%%%%%%%%%%%%%%%%%%%%%%%%%
# Match clean NAICS codes with ISIC codes Returns data with all business
# segments of companies assigned to an ISIC code and their respective revenue
# estimate
matchNaicsWithIsicCodes <- function(InputData)
{
  #################
  # Match NAICS codes with ISIC Codes starting with the most recent version of NAICS code
s
  # and the most detailed classification
  # 2017 version
  # First rename columns - simplify names of columns with NAICS and ISIC codes
  colnames(Naics2017ToIsic4)[2] <- c("NAICS")
  colnames(Naics2017ToIsic4)[5] <- c("ISIC")
  # Match
  InputData <- merge(x = InputData, y = Naics2017ToIsic4[, c(2, 5)], by = "NAICS", all.x
= TRUE)

  # In the NAICS to ISIC code mapping table, in some cases there is more than one ISIC co
de per NAICS
  # For those cases, split the revenue evenly between these codes
```

```
  # First, add a count of ISIC codes per NAICS code for each company
  InputData <- InputData %>% add_count(RIC, NAICS, name = "MultipleIsics")
  # then divide revenue by the count
  InputData$RevenuePerCode <- InputData$RevenuePerCode/InputData$MultipleIsics

  # Drop auxiliary column with Isic code count per Naics code
  InputData <- InputData[,c(-8)]

  # Not all codes have been matched so far
  # Separate those that have with those that haven't, so that we don't need to touch clea
nly matched codes in later processing
  CleanCodes <- (InputData %>% filter(!is.na(InputData$ISIC)))

  # Now there might be some multiple ISIC codes for the same company.
  # Sum these together to clean up even more
  CleanCodes <- CleanCodes %>%
    group_by(RIC, Name, PrimaryNAICS, ISIC, Sample) %>%
    summarise(RevenuePerCode = sum(RevenuePerCode))

  # Check if there was any change in total revenue
  TotalRevenue-sum(InputData$RevenuePerCode)

  # Go back to InputData and drop records that were moved to CleanCodes
  InputData <- (InputData %>% filter(is.na(InputData$ISIC)))

  # Check if there was any change in total revenue
  # This one is different from zero
  TotalRevenue-sum(InputData$RevenuePerCode)-sum(CleanCodes$RevenuePerCode)

  #########################
  # Since some of the NAICS codes used in the data are from earlier versions of the class
ification,
  # it is necessary to go back to earlier NAICS versions and map them to ISIC4 industry c
lassification.
  # This is done using 2012 and 2007 versions of NAICS. 2002 and 1997 NAICS are first map
ped to NAICS 2007,
  # before converting to ISIC4. This is because ISIC4 was introducced after 2002.
  # Importantly, only data ponts for which there is no ISIC code yet are considered itera
tively in this step.

  # 2012 version
  # First rename columns - simplify names of columns with NAICS and ISIC codes
  colnames(Naics2012ToIsic4)[1] <- c("NAICS")
  colnames(Naics2012ToIsic4)[3] <- c("ISIC2012")
  # Match
  InputData <- merge(x = InputData, y = Naics2012ToIsic4[, c(1, 3)], by = "NAICS", all.x
= TRUE)

  # In the NAICS to ISIC code mapping table, in some cases there is more than one ISIC co
de per NAICS
  # For those cases, split the revenue evenly between these codes
  # First, add a count of ISIC2012 codes per NAICS code for each company
  InputData <- InputData %>% add_count(RIC, NAICS, name = "MultipleIsics")
```

```
  # Now divide revenue from each NAICS code by the count of matched ISIC2012
  InputData$RevenuePerCode <- InputData$RevenuePerCode/InputData$MultipleIsics

  # Drop auxiliary column with Isic code count per Naics code
  InputData <- subset(InputData, select=-c(MultipleIsics))

  # Replace ISIC code NAs with ISIC codes based on 2012 NAICS
  InputData$ISIC[is.na(InputData$ISIC)] <- InputData$ISIC2012[is.na(InputData$ISIC)]

  # Drop auxiliary column with Isic codes, which were based on NAICS2012
  InputData <- subset(InputData, select=-c(ISIC2012))

  # Those codes, which have been matched with ISIC codes should be moved to CleanCodes
  AddToCleanCodes <- (InputData %>% filter(!is.na(InputData$ISIC)))
  # Drop NAICS column and append to CleanCodes
  AddToCleanCodes <- subset(AddToCleanCodes, select=-c(NAICS))
  CleanCodes <- dplyr::bind_rows(CleanCodes, AddToCleanCodes)

  # Now there might be some multiple ISIC codes for the same company.
  # Sum these together to clean up even more
  CleanCodes <- CleanCodes %>%
    group_by(RIC, Name, PrimaryNAICS, ISIC, Sample) %>%
    summarise(RevenuePerCode = sum(RevenuePerCode))

  # Go back to InputData and drop records that were moved to CleanCodes
  InputData <- (InputData %>% filter(is.na(InputData$ISIC)))

  # Check if there was any change in total revenue
  # This one is different from zero
  TotalRevenue-sum(InputData$RevenuePerCode)-sum(CleanCodes$RevenuePerCode)

  ######
  # The same procedure as for NAICS 2012 will be now used for 2007 version
  # First rename columns - simplify names of columns with NAICS and ISIC codes
  colnames(Naics2007ToIsic4)[1] <- c("NAICS")
  colnames(Naics2007ToIsic4)[3] <- c("ISIC2007")

  # Match
  InputData <- merge(x = InputData, y = Naics2007ToIsic4[, c(1, 3)], by = "NAICS", all.x
= TRUE)

  # In the NAICS to ISIC code mapping table, in some cases there is more than one ISIC co
de per NAICS
  # For those cases, split the revenue evenly between these codes
  # First, add a count of ISIC2007 codes per NAICS code for each company
  InputData <- InputData %>% add_count(RIC, NAICS, name = "MultipleIsics")

  # Now divide revenue from each NAICS code by the count of matched ISIC2012
  InputData$RevenuePerCode <- InputData$RevenuePerCode/InputData$MultipleIsics

  # Drop auxiliary column with Isic code count per Naics code
  InputData <- subset(InputData, select=-c(MultipleIsics))

  # Replace ISIC code NAs with ISIC codes based on 2007 NAICS
```

```
  InputData$ISIC[is.na(InputData$ISIC)] <- InputData$ISIC2007[is.na(InputData$ISIC)]

  # Drop auxiliary column with Isic codes, which were based on NAICS2007
  InputData <- subset(InputData, select=-c(ISIC2007))

  # Those codes, which have been matched with ISIC codes should be moved to CleanCodes
  AddToCleanCodes <- (InputData %>% filter(!is.na(InputData$ISIC)))
  # Drop NAICS column and append to CleanCodes
  AddToCleanCodes <- subset(AddToCleanCodes, select=-c(NAICS))
  CleanCodes <- dplyr::bind_rows(CleanCodes, AddToCleanCodes)

  # Now there might be some multiple ISIC codes for the same company.
  # Sum these together to clean up even more
  CleanCodes <- CleanCodes %>%
    group_by(RIC, Name, PrimaryNAICS, ISIC, Sample) %>%
    summarise(RevenuePerCode = sum(RevenuePerCode))

  # Go back to InputData and drop records that were moved to CleanCodes
  InputData <- (InputData %>% filter(is.na(InputData$ISIC)))

  # Check if there was any change in total revenue
  TotalRevenue-sum(InputData$RevenuePerCode)-sum(CleanCodes$RevenuePerCode)

  #####
  # For older versions of NAICS there is no direct concordance table that would map them
to ISIC codes revision 4.
  # Thus, 2002 NAICS are mapped first to NAICS 2007. After that 1997 NAICS are updated fi
rst to 2002, then to 2007 version
  # First rename columns - simplify names of columns with NAICS and ISIC codes
  colnames(Naics2002To2007)[1] <- c("NAICS")
  colnames(Naics2002To2007)[3] <- c("NAICS2007")

  # Match 2002 NAICS with 2007 NAICS
  InputData <- merge(x = InputData, y = Naics2002To2007[, c(1, 3)], by = "NAICS", all.x =
TRUE)

  # Since one NAICS2002 can be mapped to multiple NAICS2007, revenue for this code needs
to be split
  # First, add a count of NAICS2007 codes per NAICS code for each company
  InputData <- InputData %>% add_count(RIC, NAICS, name = "MultipleNaics")

  # Divide revenue from each NAICS code by the count of matched NAICS2007 codes
  InputData$RevenuePerCode <- InputData$RevenuePerCode/InputData$MultipleNaics

  # Check if there was any change in total revenue
  TotalRevenue-sum(InputData$RevenuePerCode)-sum(CleanCodes$RevenuePerCode)

  # Those NAICS codes which were updated to 2007 version can be separated, so that we can
deal with the remaining ones
  NAICSUpdatedTo2007 <- InputData
  NAICSUpdatedTo2007 <- (NAICSUpdatedTo2007 %>% filter(!is.na(NAICSUpdatedTo2007$NAICS200
7)))

  # Go back to InputData and drop records that were moved to NAICSUpdatedTo2007
```

```
  InputData <- (InputData %>% filter(is.na(InputData$NAICS2007)))

  # Drop auxiliary columns with NAICS2007 and their count
  InputData <- subset(InputData, select=-c(NAICS2007, MultipleNaics))

  # Start working with 1997 NAICS
  # First rename columns - simplify names of columns with NAICS and ISIC codes
  colnames(Naics1997To2002)[1] <- c("NAICS")
  colnames(Naics1997To2002)[3] <- c("NAICS2002")

  # Match 1997 NAICS with 2002 NAICS
  InputData <- merge(x = InputData, y = Naics1997To2002[, c(1, 3)], by = "NAICS", all.x =
TRUE)

  ########### CHECK POINT ###########
  # By now all NAICS codes in InputData should be matched with NAICS2002 codes.
  # In other words, check that there are no NAs in NAICS2002 column.
  # If there are, then there is some issue with the data, as all versions of NAICS have b
een already iterated through.
  ########### ###########

  sum(is.na(InputData$NAICS2002))

  # Since one NAICS1997 can be mapped to multiple NAICS2002, revenue for this code needs
to be split
  # First, add a count of NAICS2007 codes per NAICS code for each company
  InputData <- InputData %>% add_count(RIC, NAICS, name = "MultipleNaics")

  # Divide revenue from each NAICS code by the count of matched NAICS2007 codes
  InputData$RevenuePerCode <- InputData$RevenuePerCode/InputData$MultipleNaics

  # Check if total revenue has changed
  TotalRevenue-sum(InputData$RevenuePerCode)-sum(CleanCodes$RevenuePerCode)-sum(NAICSUpda
tedTo2007$RevenuePerCode)

  # Since all NAICS codes are matched with 2002 version, we can get rid of 1997 NAICS cod
es and use only 2002 now.
  # Also, it is important to recognize that in some cases multiple 1997 NAICS were integr
ated into a single NAICS2002.
  # This means, that records that belong to the same company and NAICS2002 code should be
intergrated
  InputData <- InputData %>%
    group_by(RIC, Name, PrimaryNAICS, NAICS2002, Sample) %>%
    summarise(RevenuePerCode = sum(RevenuePerCode))

  # Rename NAICS 2002 to NAICS
  colnames(InputData)[4] <- c("NAICS")

  # Match the codes which were updated to 2002 NAICS with 2007 NAICS
  InputData <- merge(x = InputData, y = Naics2002To2007[, c(1, 3)], by = "NAICS", all.x =
TRUE)

  # Since one NAICS2002 can be mapped to multiple NAICS2007, revenue for this code needs
to be split
```

```
  # First, add a count of NAICS2007 codes per NAICS code for each company
  InputData <- InputData %>% add_count(RIC, NAICS, name = "MultipleNaics")

  # Divide revenue from each NAICS code by the count of matched NAICS2007 codes
  InputData$RevenuePerCode <- InputData$RevenuePerCode/InputData$MultipleNaics

  # Check if total revenue has changed
  TotalRevenue-sum(InputData$RevenuePerCode)-sum(CleanCodes$RevenuePerCode)-sum(NAICSUpda
tedTo2007$RevenuePerCode)

  # Now InputData can be combined with those that were already earlier updated to NAICS 2
007 version
  NAICSUpdatedTo2007 <- dplyr::bind_rows(NAICSUpdatedTo2007, InputData)

  # Now there might be some multiple NAICS2007 codes for the same company.
  # Sum these together to clean up even more
  # Now ISIC code is dropped - there were only NAs here
  NAICSUpdatedTo2007 <- NAICSUpdatedTo2007 %>%
    group_by(RIC, Name, PrimaryNAICS, NAICS2007, Sample) %>%
    summarise(RevenuePerCode = sum(RevenuePerCode))

  # Check if total revenue has changed
  TotalRevenue-sum(CleanCodes$RevenuePerCode)-sum(NAICSUpdatedTo2007$RevenuePerCode)

  # Now it is possible to match NAICS codes, which were updated to 2007 version, with ISI
C codes
  # Match
  NAICSUpdatedTo2007 <- merge(x = NAICSUpdatedTo2007, y = Naics2007ToIsic4[, c(1, 3)], by
.x = "NAICS2007", by.y = "NAICS", all.x = TRUE)

  # In the NAICS to ISIC code mapping table, in some cases there is more than one ISIC co
de per NAICS
  # For those cases, split the revenue evenly between these codes
  # First, add a count of ISIC2007 codes per NAICS code for each company
  NAICSUpdatedTo2007 <- NAICSUpdatedTo2007 %>% add_count(RIC, NAICS2007, name = "Multiple
Isics")

  # Now divide revenue from each NAICS code by the count of matched ISIC2012
  NAICSUpdatedTo2007$RevenuePerCode <- NAICSUpdatedTo2007$RevenuePerCode/NAICSUpdatedTo20
07$MultipleIsics

  # Check if total revenue has changed
  TotalRevenue-sum(CleanCodes$RevenuePerCode)-sum(NAICSUpdatedTo2007$RevenuePerCode)

  # Drop auxiliary column with Isic code count per Naics code
  NAICSUpdatedTo2007 <- subset(NAICSUpdatedTo2007, select=-c(MultipleIsics))

  # Rename column ISIC2007 to ISIC
  names(NAICSUpdatedTo2007)[names(NAICSUpdatedTo2007) == "ISIC2007"] <- "ISIC"

  # Drop column with NAICS codes, as they are not needed any more
  NAICSUpdatedTo2007 <- subset(NAICSUpdatedTo2007, select=-c(NAICS2007))

  # ISIC codes, which were matched based on NAICS update to 2007, can now be merged with
```

```
CleanCodes
  CleanCodes <- dplyr::bind_rows(CleanCodes, NAICSUpdatedTo2007)

  # Check if total revenue has changed
  TotalRevenue-sum(CleanCodes$RevenuePerCode)

  # Now there might be some multiple ISIC codes for the same company.
  # Sum these together to clean up even more
  CleanCodes <- CleanCodes %>%
    group_by(RIC, Name, ISIC, Sample) %>%
    summarise(RevenuePerCode = sum(RevenuePerCode))

  # Check if there was any change in total revenue
  # This one is different from zero
  TotalRevenue-sum(CleanCodes$RevenuePerCode)

  # Return the final matching
  return(CleanCodes)
}

#%%%%%%%%%%%%%%%%%%%%%%%%%%%%%%%%%%%%%%%%%%%%%%%%%%%%%%%%%%%%%%%%%%%%%%%%%%%%%%%%
#%%%%%%%%%%%%%%%%%% Steps 3 and 4 of the method %%%%%%%%%%%%%%%%%%%%%%%%%%%%%%%%%%%
#%%%%%%%%%%%%%%%%%%%%%%%%%%%%%%%%%%%%%%%%%%%%%%%%%%%%%%%%%%%%%%%%%%%%%%%%%%%%%%%%
# Match each segment ISIC code with Digital Intensity score and weight these
# scores with revenue to calculate revenue weighted Digital Intensity on a
# company-level
matchIsicWithDigitalIntensity <- function(CleanCodes)
{
  #########################
  # By now all NAICS codes should have been mapped to ISIC codes.
  # This means that further analysis based on ISIC codes can be performed
  # Match digital intensity with the ISIC codes
  # Create a column with first two digits of ISIC code
  #########################
  CleanCodes$ShortISIC <- str_sub(CleanCodes$ISIC, 1,2)

  # Match short ISIC codes with digital intensity. If there are any ShortISIC
  # codes included in CleanCodes, which don't have any Digital Intensity score
  # assigned, then there will be "numeric(0)" values. These, need to be replaced
  # with NAs.
  CleanCodes$DigitalIntensity <- apply(CleanCodes, 1, function(x) {
    score <- QuartileComparison$DiScore[
      as.numeric(QuartileComparison$ISIC_High) >= as.numeric(x[6]) &
        as.numeric(QuartileComparison$ISIC_Low) <= as.numeric(x[6])]

    if(length(score) == 0){
      return(NA)
    } else {
      return(score)
    }
  }
  )


  # Count any business segments that might be missing Digital Intensity score.
```

```
  # This can happen if ISIC to DI mapping table doesn't include some ISIC codes
  message("There are ", sum(is.na(CleanCodes$DigitalIntensity)), " missing Digital Intens
ity scores in the final data.")
  message("If needed, inspect manually, which ISIC codes are missing Digital Intensity sc
ores.")
  message("For now, these will be deleted manually, but you might want to revise the data
and re-run the code.")

  # Drop rows with NAs
  # CleanCodes <- CleanCodes[complete.cases(CleanCodes), ]

  # If it is acceptable, drop rows with Digital Intensity being NA
  CleanCodes <- CleanCodes %>% filter(!is.na(DigitalIntensity))

  #######
  # Calculate weighted score for digital intensity for each company
  # Calculate product of digital intensity and revenue
  CleanCodes$RevXDigi <- CleanCodes$RevenuePerCode*CleanCodes$DigitalIntensity

  # For each company calculate revenue weighted digital intensity score
  CompanyDI <- CleanCodes %>%
    group_by(RIC, Name, Sample) %>%
    summarise(WeightedDigitalIntensity = sum(RevXDigi)/sum(RevenuePerCode))

  return(CompanyDI)
}

#%%%%%%%%%%%%%%%%%%%%%%%%%%%%%%%%%%%%%%%%%%%%%%%%%%%%%%%%%%%%%%%%%%%%%%%%%%%%%%%%%
#%%%%%%%%%%%%%%%%%% Step 5 of the method %%%%%%%%%%%%%%%%%%%%%%%%%%%%%%%%%%%%%%%%%
#%%%%%%%%%%%%%%%%%%%%%%%%%%%%%%%%%%%%%%%%%%%%%%%%%%%%%%%%%%%%%%%%%%%%%%%%%%%%%%%%%
# Assign company-level Digital Intensity scores into one of three levels of
# digital intensity.
assignDigitalIntensity <- function(CompanyDI)
{
  CompanyDI$DigitalIntensityLevel <- 1
  CompanyDI$DigitalIntensityLevel[CompanyDI$WeightedDigitalIntensity > quantile(OecdRank$
DiScore, 1/3) &
                                    CompanyDI$WeightedDigitalIntensity <= quantile(OecdRa
nk$DiScore, 2/3)] <- 2
  CompanyDI$DigitalIntensityLevel[CompanyDI$WeightedDigitalIntensity > quantile(OecdRank$
DiScore, 2/3)] <- 3

  return(CompanyDI)
}

##########################################################
# Process the data with information on business segment-level
##########################################################

# Add a column marking the sample
USData$Sample = "US"
NonUSData$Sample = "Non-US"

# Combine data from both samples
```

```
InputData <- dplyr::bind_rows(USData, NonUSData)

# Process the data to get Digital Intensity for each company
InputData <- cleanData(InputData)

## Warning in cleanData(InputData): NAs introduced by coercion

## Warning: Expected 4 pieces. Missing pieces filled with `NA` in 5310 rows [1, 2,
## 3, 4, 5, 6, 7, 8, 9, 10, 11, 12, 13, 14, 15, 16, 17, 18, 19, 20, ...].

## Warning in cleanData(InputData): NAs introduced by coercion

## Check if there was any change in total revenue

## Difference is 0

CleanCodes <- matchNaicsWithIsicCodes(InputData)
CompanyDI <- matchIsicWithDigitalIntensity(CleanCodes)

## There are 2 missing Digital Intensity scores in the final data.

## If needed, inspect manually, which ISIC codes are missing Digital Intensity scores.

## For now, these will be deleted manually, but you might want to revise the data and re-
run the code.

CompanyDI <- assignDigitalIntensity(CompanyDI)


##########################################################
# Analysis of the results
##########################################################

# Join tables with Digital Intensity estimates based on segment-level data with
# the initial input data to get NAICS primary industry codes.
FinalData <- unique(merge(x = CompanyDI, y = InputData[, c(1, 3)], by = "RIC", all.x = TR
UE))


# Rename columns to make them shorter
colnames(FinalData)[4] = "SegDI"
colnames(FinalData)[5] = "SegDiLevel"

# Add Segment Digital Intensity Level as text
FinalData$SegDiLevelTxt <- "1 - Low"
FinalData$SegDiLevelTxt[FinalData$SegDiLevel == 2] <- "2 - Mid"
FinalData$SegDiLevelTxt[FinalData$SegDiLevel == 3] <- "3 - High"

# Create PrimarySector code based on primary NAICS code
FinalData$PrimSector <- str_sub(FinalData$PrimaryNAICS, 1,2)

#####################
# Plot 1
# Plot giving an overview of the sample data - count by segment for each sample
FinalData %>% count(Sample, PrimSector) %>%
  ggplot(aes(x = n, y = PrimSector)) +
  geom_col() +
```

```r
  ylab( "Primary sector code based on NAICS" ) +
  xlab( "Count of companies" ) +
  coord_flip() +
  facet_grid(cols = vars(Sample))
```

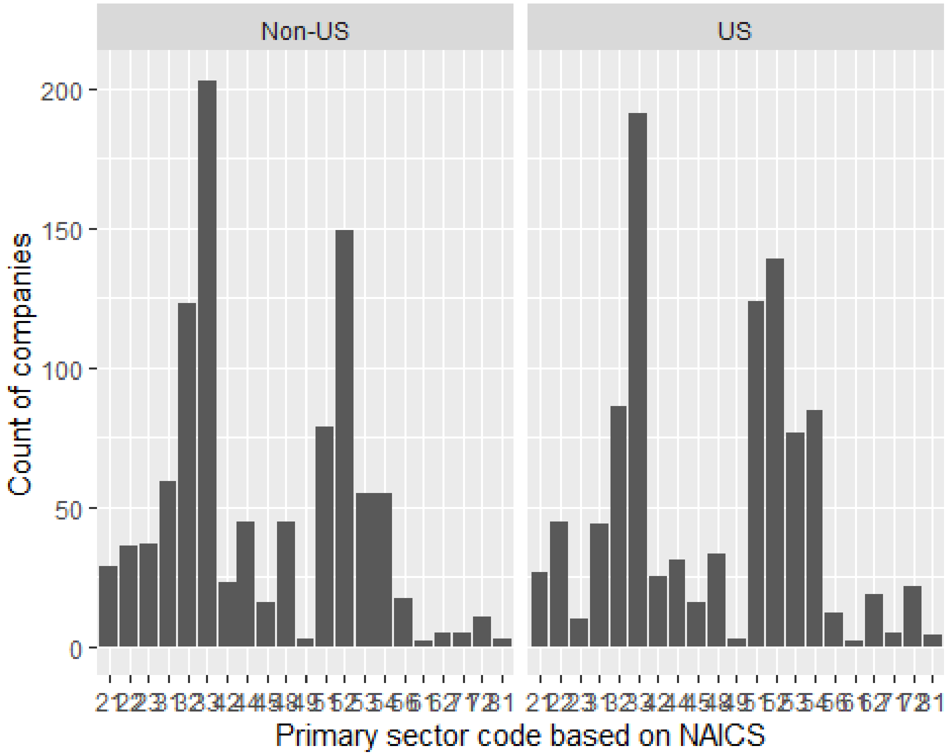


```r
####################
# Plot 2
# Scatter plot showing an overview of the main results
ggplot(data = FinalData, aes(x = PrimSector,
                             y = SegDI,
                             color= SegDiLevelTxt,
                             shape=SegDiLevelTxt)) +
  geom_point(size=3) +
  scale_shape_manual(values=c(8, 1, 2)) +
  scale_color_manual(values=c('deepskyblue1','springgreen4', 'red')) +
  facet_grid(cols = vars(Sample)) +
  xlab( "Primary sector code based on NAICS" ) +
  ylab( "Digital intensity score weighted by business segment revenue" ) +
  theme(legend.position="top",
        panel.grid.major.y =element_blank(),
        panel.grid.minor.y =element_blank(),
        axis.text.y=element_blank(),
        axis.ticks.y=element_blank()) +
  labs(color = "Estimated digital intensity of a company",
       shape = "Estimated digital intensity of a company")
```

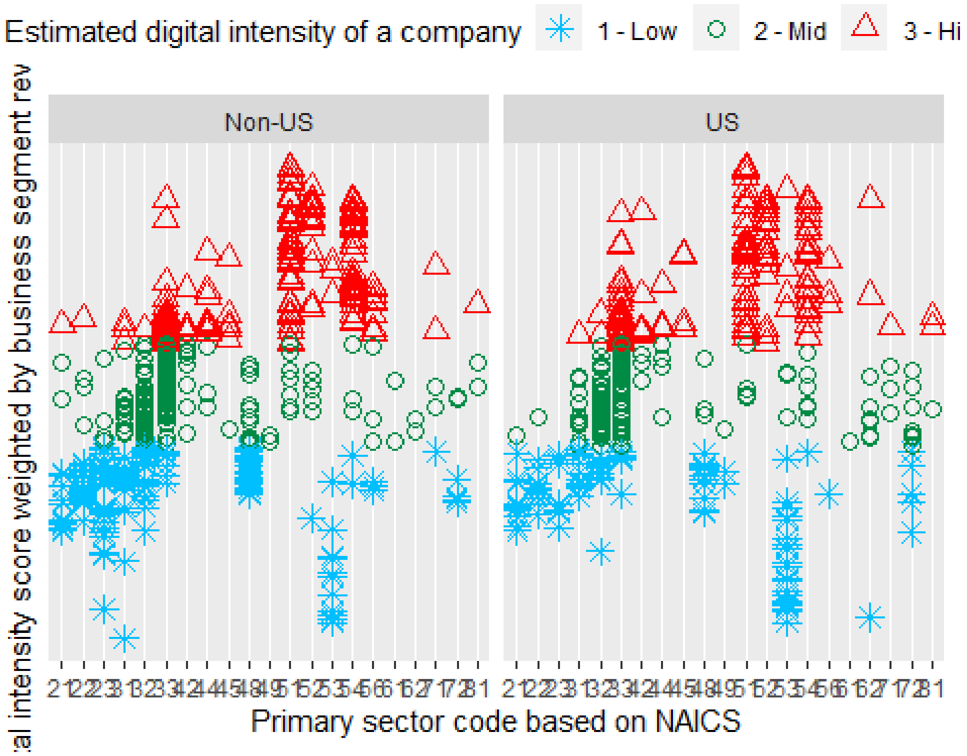


```
#########################################################
# Use equivalent samples and compare results when business segment industry codes
# are used versus if only primary industry codes are used
#########################################################

# Create a new data frame to hold only the records where business segment
# industry codes are available
EquivalentInputData <- dplyr::bind_rows(USData, NonUSData)
EquivalentInputData <- EquivalentInputData[!is.na(EquivalentInputData$`Segment Code`),]

####### Equivalent sampel - Business segment data
# Estimate firm-level digital intensity using business segment industry codes
InputDataSeg <- cleanData(EquivalentInputData)

## Warning in cleanData(EquivalentInputData): NAs introduced by coercion

## Warning: Expected 4 pieces. Missing pieces filled with `NA` in 4782 rows [1, 2,
## 3, 4, 5, 6, 7, 8, 9, 10, 11, 12, 13, 14, 15, 16, 17, 18, 19, 20, ...].

## Warning in cleanData(EquivalentInputData): NAs introduced by coercion

## Check if there was any change in total revenue

## Difference is 0

# The previous 2 step have eliminated companies with missing business segment
# industry codes, with the exception of one case. Companies, which had non-NA
# segment code data, but didn't actually include industry codes, but some other
# text in that field. We need to find these now, so that they can be eliminated.
# This way we will have a sample of companies, which for all cases has business
# segment industry codes. These companies can now be identified by an arbitrary
# revenue of 1 and primary and segment industry code being equivalent.
ToBeDropped <- InputDataSeg %>%
```

```
  filter(RevenuePerCode == 1 & PrimaryNAICS == NAICS)

# Drop these companies
InputDataSeg <- InputDataSeg %>%
  filter(!RIC %in% ToBeDropped$RIC)

# Do rest of the processing and estimate digital intensity of the firms included
# in the sample
CleanCodesSeg <- matchNaicsWithIsicCodes(InputDataSeg)
CompanyDISeg <- matchIsicWithDigitalIntensity(CleanCodesSeg)

## There are 2 missing Digital Intensity scores in the final data.

## If needed, inspect manually, which ISIC codes are missing Digital Intensity scores.

## For now, these will be deleted manually, but you might want to revise the data and re-
run the code.

CompanyDISeg <- assignDigitalIntensity(CompanyDISeg)

####### Equivalent sampel - Primary industry code only data
# Start with the same data
InputDataPrimaryIndustry <- EquivalentInputData %>%
  filter(!RIC %in% ToBeDropped$RIC)

# Assign NA to segment name, code and revenue, so that the processing will happen on comp
any level using primary industry code only
InputDataPrimaryIndustry$`Segment Name` = NA
InputDataPrimaryIndustry$`Segment Code` = NA
InputDataPrimaryIndustry$`Business Total Revenues (Calculated)` = NA

# Process the data to get Digital Intensity for each company
InputDataPrimaryIndustry <- cleanData(InputDataPrimaryIndustry)

## Warning: Expected 4 pieces. Missing pieces filled with `NA` in 1464 rows [1, 2,
## 3, 4, 5, 6, 7, 8, 9, 10, 11, 12, 13, 14, 15, 16, 17, 18, 19, 20, ...].

## Check if there was any change in total revenue

## Difference is 0

CleanCodesPrimaryIndustry <- matchNaicsWithIsicCodes(InputDataPrimaryIndustry)
CompanyDIPrimaryIndustry <- matchIsicWithDigitalIntensity(CleanCodesPrimaryIndustry)

## There are 0 missing Digital Intensity scores in the final data.

## If needed, inspect manually, which ISIC codes are missing Digital Intensity scores.

## For now, these will be deleted manually, but you might want to revise the data and re-
run the code.

CompanyDIPrimaryIndustry <- assignDigitalIntensity(CompanyDIPrimaryIndustry)

##################### Reformat the final data for comparison
# Join tables with Digital Intensity estimates based on segment-level data and
# primary industry. Add also NAICS primary industry.
FinalData <- merge(x = CompanyDISeg, y = CompanyDIPrimaryIndustry[, c(1, 4, 5)], by = "RI
C", all.x = TRUE)
```

```
FinalData <- merge(x = FinalData, y = InputDataPrimaryIndustry[, c(1, 3)], by = "RIC", al
l.x = TRUE)


# Rename columns to make them shorter
colnames(FinalData)[4] = "SegDI"
colnames(FinalData)[5] = "SegDiLevel"
colnames(FinalData)[6] = "PrimDI"
colnames(FinalData)[7] = "PrimDiLevel"

# Add Segment Digital Intensity Level as text
FinalData$SegDiLevelTxt <- "1 - Low"
FinalData$SegDiLevelTxt[FinalData$SegDiLevel == 2] <- "2 - Mid"
FinalData$SegDiLevelTxt[FinalData$SegDiLevel == 3] <- "3 - High"

# Create PrimarySector code based on primary NAICS code
FinalData$PrimSector <- str_sub(FinalData$PrimaryNAICS, 1,2)


####################
# Plot 3 Plot an overview of agreement between digital intensity based on
# business segment revenue weighted scores and those calculated based on primary
# industry only
FinalData %>% group_by(Sample) %>%
  add_count(Sample, name = "countSample") %>%
  add_count(Sample, SegDiLevel, PrimDiLevel, name = "caseCount") %>%
  mutate(Percentage = caseCount / countSample) %>%
  group_by(Sample, SegDiLevel, PrimDiLevel, Percentage) %>%
  summarise() %>%
  ggplot(aes(x = as.factor(SegDiLevel),
             y = as.factor(PrimDiLevel),
             size = Percentage,
             label = paste0(round(100 * Percentage, 1), "%"))) +
  geom_count(color = "deepskyblue1") +
  ylab( "Digital intensity based on primary industry" ) +
  xlab( "Digital intensity based on industry weighted by business segment revenue" ) +
  facet_grid(cols = vars(Sample)) +
  scale_size_area(max_size = 40) +
  geom_label(size = 4, nudge_y = 0.33) +
  theme(legend.position = "none",
        panel.grid.minor.y =element_blank(),
        panel.grid.minor.x =element_blank()) +
  scale_x_discrete(labels=c("0" = "", "1" = "Low","2" = "Mid","3" = "High","4" = "")) +
  scale_y_discrete(labels=c("0" = "", "1" = "Low","2" = "Mid","3" = "High","4" = ""))
```

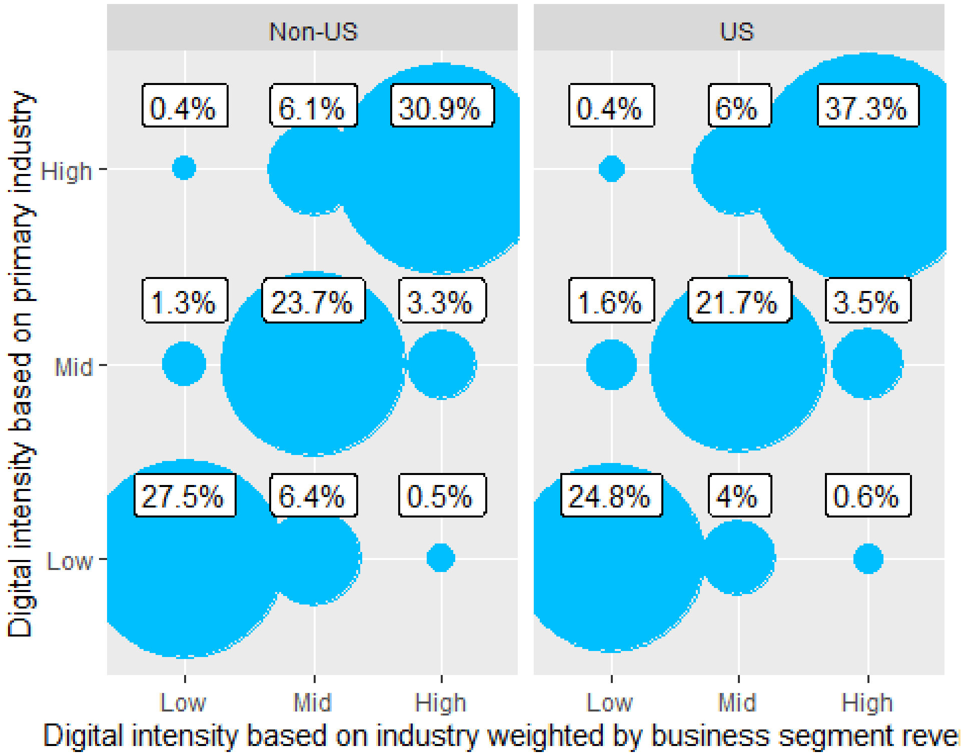


```
# Estimate Cohen's kappa for the two approaches
kappa2(FinalData[FinalData$Sample == "Non-US",c(5,7)])

##  Cohen's Kappa for 2 Raters (Weights: unweighted)
##
##  Subjects = 786
##    Raters = 2
##     Kappa = 0.731
##
##         z = 29.2
##   p-value = 0

kappa2(FinalData[FinalData$Sample == "US",c(5,7)])

##  Cohen's Kappa for 2 Raters (Weights: unweighted)
##
##  Subjects = 678
##    Raters = 2
##     Kappa = 0.752
##
##         z = 27.6
##   p-value = 0

# Analyze the level of agreement between the two approaches
agree(FinalData[FinalData$Sample == "Non-US",c(5,7)])

##  Percentage agreement (Tolerance=0)
##
##  Subjects = 786
##    Raters = 2
##   %-agree = 82.1

agree(FinalData[FinalData$Sample == "US",c(5,7)])
```

```
##  Percentage agreement (Tolerance=0)
##
##  Subjects = 678
##    Raters = 2
##   %-agree = 83.8
```

```
# Additionally, estimate confidence interval for the percentage agreement - this
# is done using bootstrap method. Since bootstrap takes in data in vector format
# and our data is more complex, there is a need to create a walk-around
# solution.
xdata <- FinalData[FinalData$Sample == "Non-US",c(5,7)]
n <- length(xdata[,1])
theta <- function(x,xdata){ agree(xdata[x,])$value }
lowBand <- function(x){quantile(x, .025)}
highBand <- function(x){quantile(x, .975)}
results <- bootstrap(1:n,5000,theta,xdata)
lowBand(results$thetastar)
```

```
##     2.5%
## 79.26209
```

```
highBand(results$thetastar)
```

```
##    97.5%
## 84.73282
```

```
xdata <- FinalData[FinalData$Sample == "US",c(5,7)]
n <- length(xdata[,1])
results <- bootstrap(1:n,5000,theta,xdata)
lowBand(results$thetastar)
```

```
##     2.5%
## 80.97345
```

```
highBand(results$thetastar)
```

```
##    97.5%
## 86.43068
```

```
#####################
# End of analysis
#####################
```
